# Supplementary material for: Ten Plastomes of Crassula (Crassulaceae) and Phylogenetic Implications
Source: Biology (Basel). 2022 Dec 7;11(12):1779. doi: 10.3390/biology11121779 (PMC9775174; doi:10.3390/biology11121779)
Supplement: Supplementary file 1 [file biology-11-01779-s001.zip › Supplements/Figure S1.pdf]

accD:

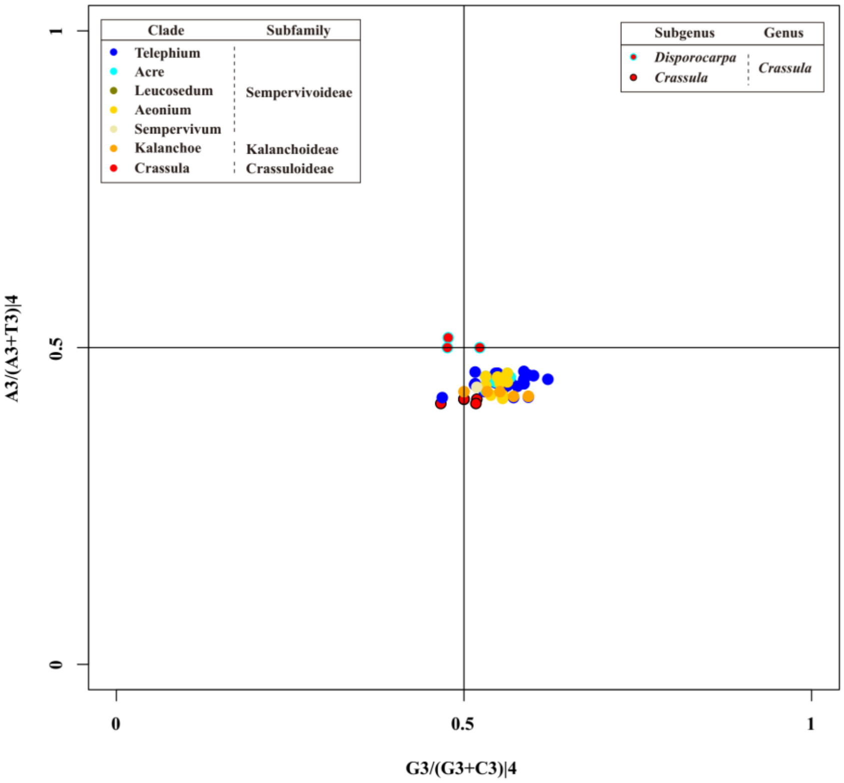
Clade
Subfamily
Telephium
Acre
Leucosedum
Aeonium
Sempervivum
Kalanchoe
Crassula
Sempervivoideae
Kalanchoideae
Crassuloideae
Subgenus
Genus
Disporocarpa
Crassula
Crassula
A3/(A3+T3)|4
G3/(G3+C3)|4
0
0.5
1
0
0.5
1

atpA:

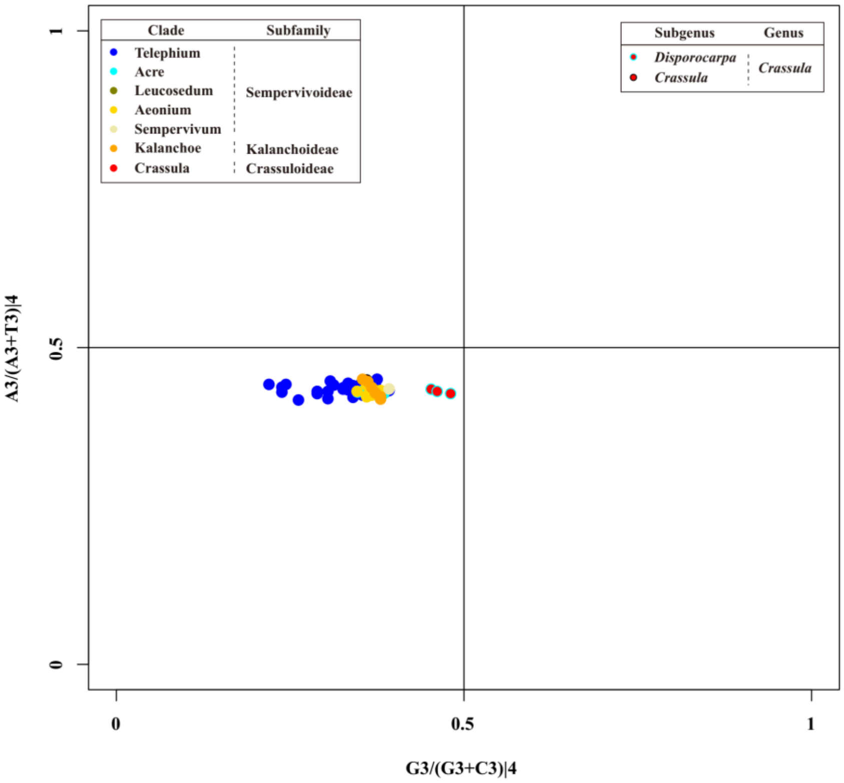
Clade
Subfamily
Telephium
Acre
Leucosedum
Aeonium
Sempervivum
Kalanchoe
Crassula
Sempervivoideae
Kalanchoideae
Crassuloideae
Subgenus
Genus
Disporocarpa
Crassula
Crassula
A3/(A3+T3)|4
G3/(G3+C3)|4
0
0.5
1
0
0.5
1

atpB:

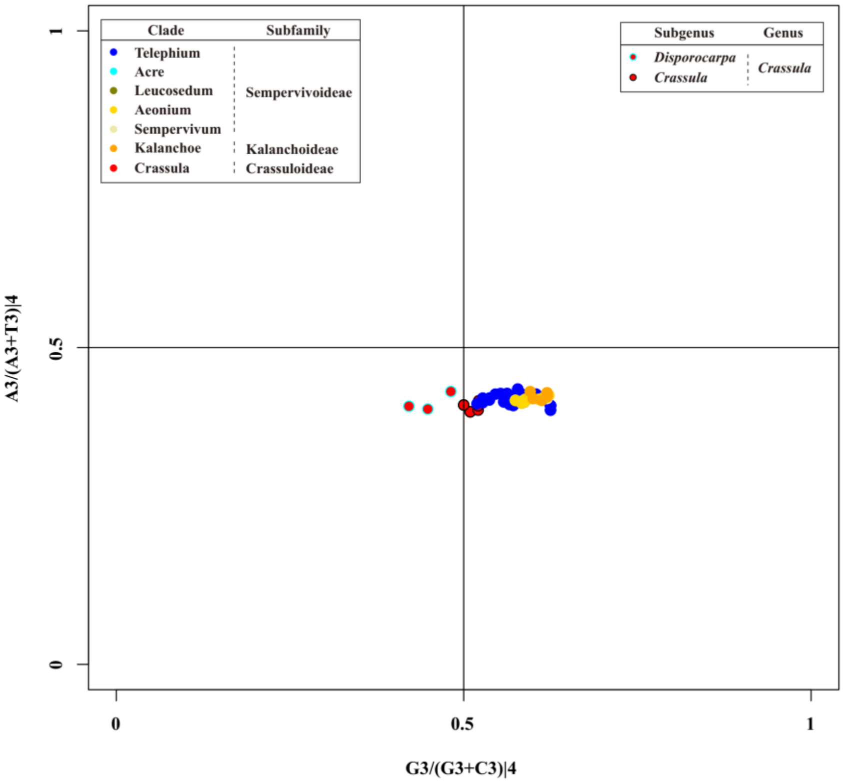

Clade
Subfamily
Telephium
Acre
Leucosedum
Aeonium
Sempervivum
Kalanchoe
Crassula
Sempervivoideae
Kalanchoideae
Crassuloideae
Subgenus
Genus
Disporocarpa
Crassula
Crassula
A3/(A3+T3)|4
G3/(G3+C3)|4
0
0.5
1

atpE:

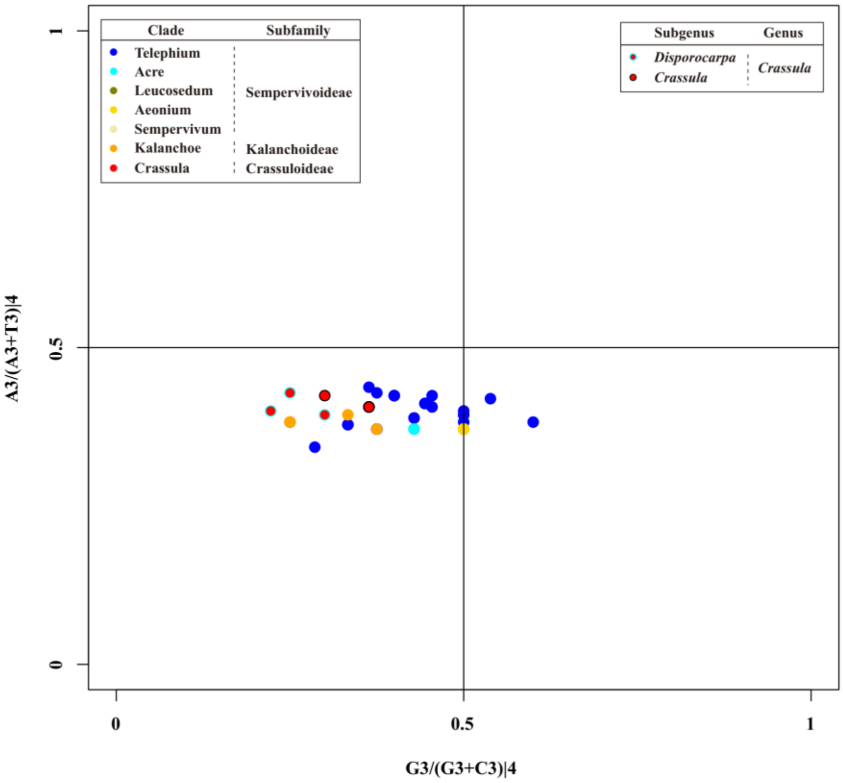

Clade
Subfamily
Telephium
Acre
Leucosedum
Aeonium
Sempervivum
Kalanchoe
Crassula
Sempervivoideae
Kalanchoideae
Crassuloideae
Subgenus
Genus
Disporocarpa
Crassula
Crassula
A3/(A3+T3)|4
G3/(G3+C3)|4
0
0.5
1

atpF:

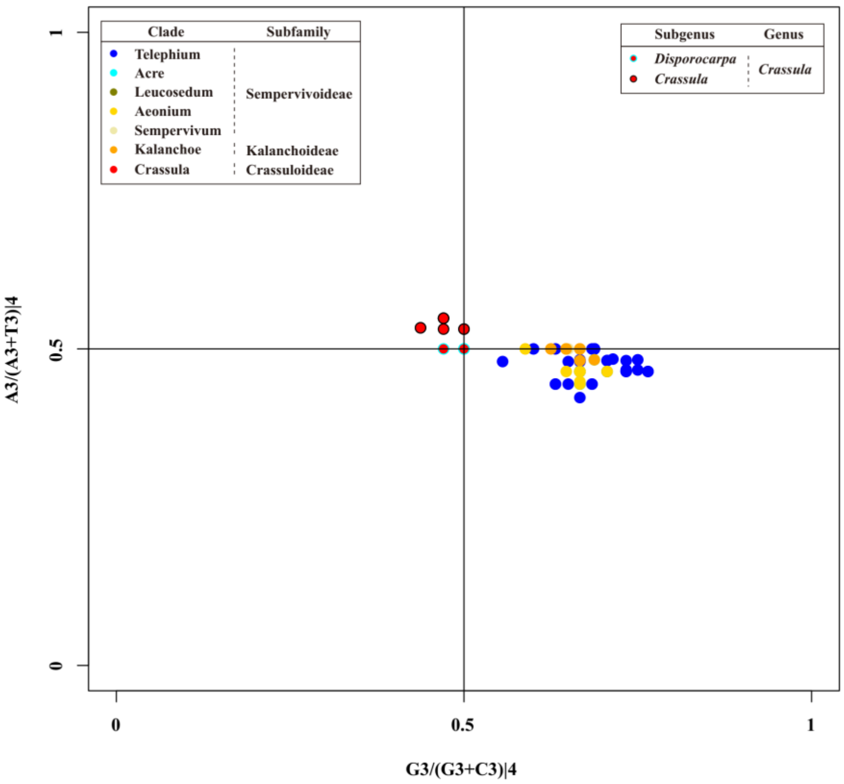

Clade
Subfamily
Telephium
Acre
Leucosedum
Aeonium
Sempervivum
Sempervivoideae
Kalanchoe
Kalanchoideae
Crassula
Crassuloideae
Subgenus
Genus
Disporocarpa
Crassula
Crassula
A3/(A3+T3)|4
G3/(G3+C3)|4
0
0.5
1

atpI:

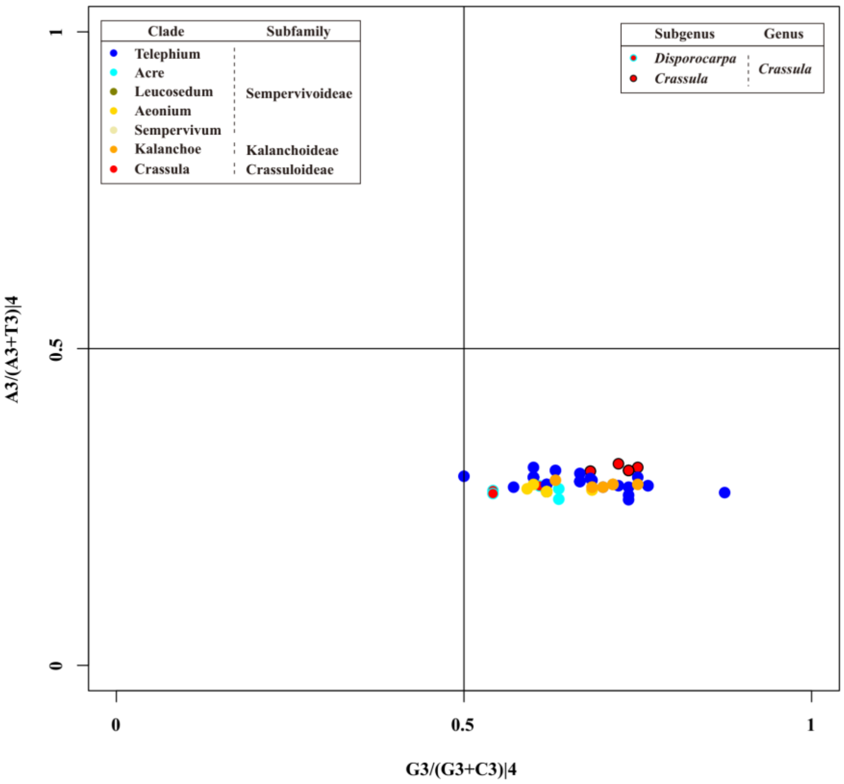

Clade
Subfamily
Telephium
Acre
Leucosedum
Aeonium
Sempervivum
Sempervivoideae
Kalanchoe
Kalanchoideae
Crassula
Crassuloideae
Subgenus
Genus
Disporocarpa
Crassula
Crassula
A3/(A3+T3)|4
G3/(G3+C3)|4
0
0.5
1

ccsA:

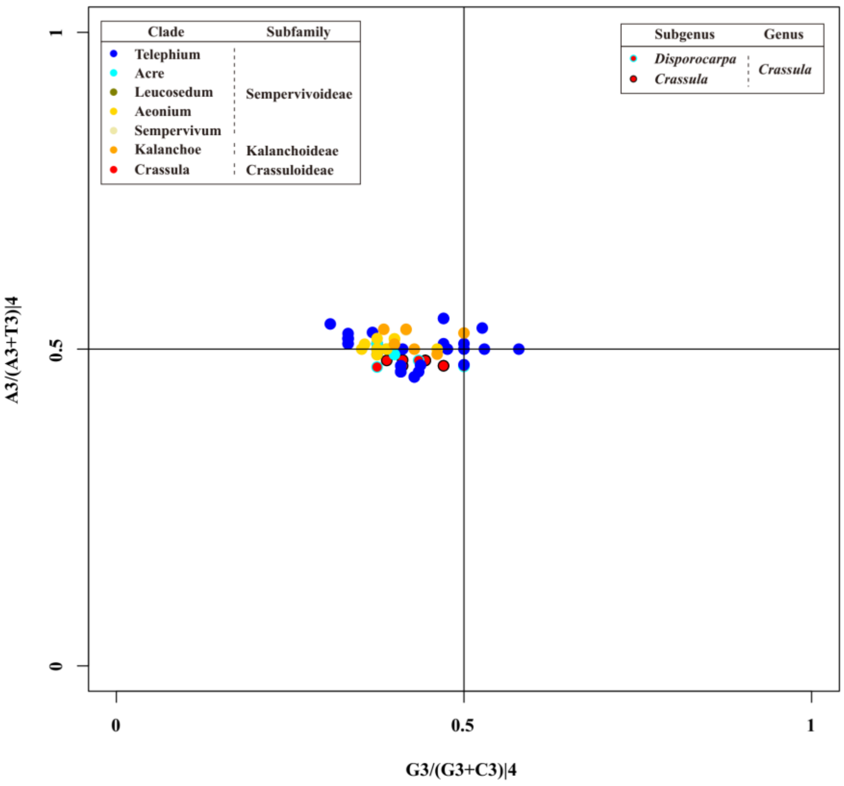
Clade
Subfamily
Telephium
Acre
Leucosedum
Aeonium
Sempervivum
Kalanchoe
Crassula
Sempervivoideae
Kalanchoideae
Crassuloideae
Subgenus
Genus
Disporocarpa
Crassula
Crassula
A3/(A3+T3)|4
G3/(G3+C3)|4
0
0.5
1
0
0.5
1

cemA:

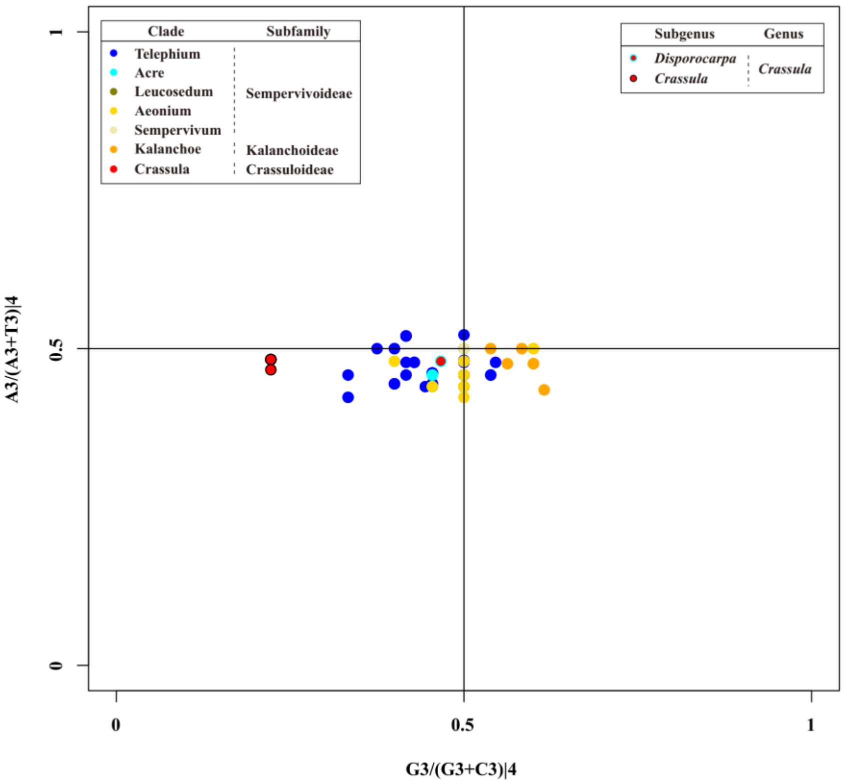
Clade
Subfamily
Telephium
Acre
Leucosedum
Aeonium
Sempervivum
Kalanchoe
Crassula
Sempervivoideae
Kalanchoideae
Crassuloideae
Subgenus
Genus
Disporocarpa
Crassula
Crassula
A3/(A3+T3)|4
G3/(G3+C3)|4
0
0.5
1
0
0.5
1

clpP:

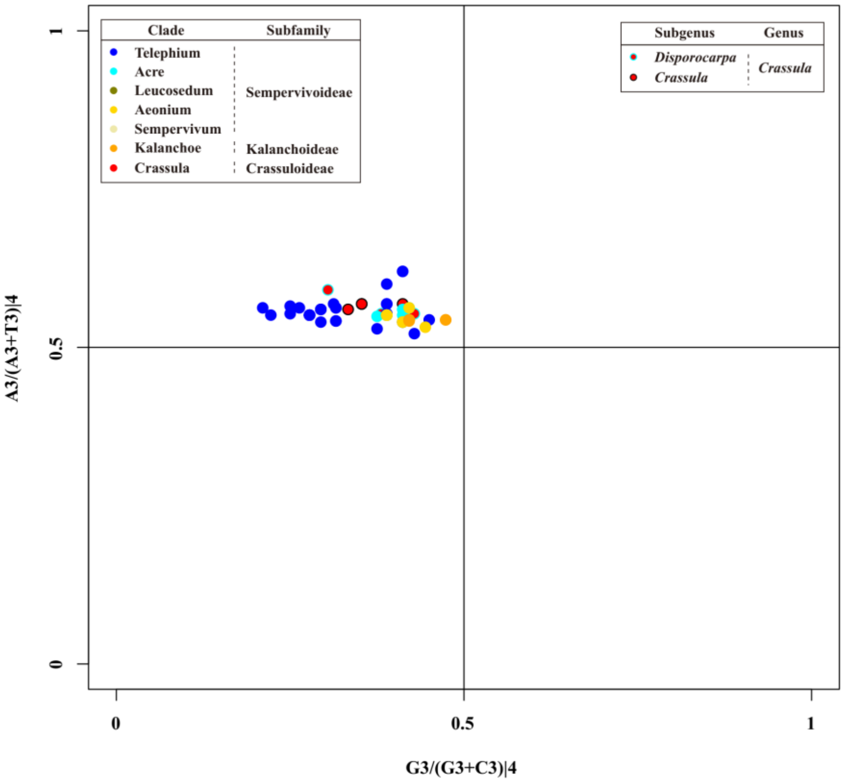
Clade
Subfamily
Telephium
Acre
Leucosedum
Aeonium
Sempervivum
Sempervivoideae
Kalanchoe
Kalanchoideae
Crassula
Crassuloideae
Subgenus
Genus
Disporocarpa
Crassula
Crassula
1
0.5
0
A3/(A3+T3)|4
0
0.5
1
G3/(G3+C3)|4

ndhA:

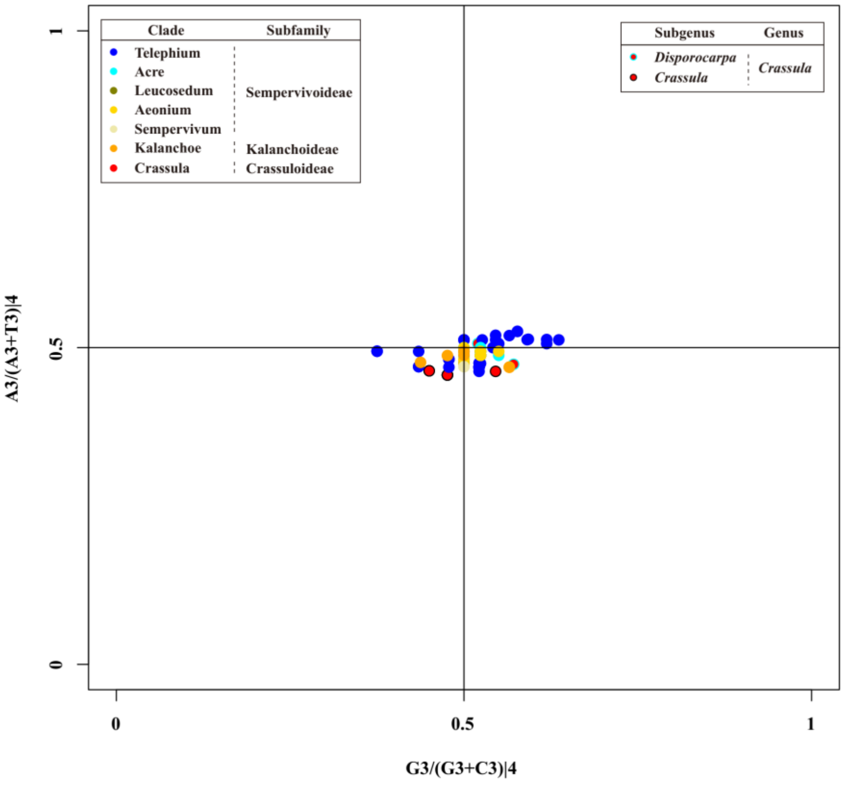
Clade
Subfamily
Telephium
Acre
Leucosedum
Aeonium
Sempervivum
Sempervivoideae
Kalanchoe
Kalanchoideae
Crassula
Crassuloideae
Subgenus
Genus
Disporocarpa
Crassula
Crassula
1
0.5
0
A3/(A3+T3)|4
0
0.5
1
G3/(G3+C3)|4

ndhB:

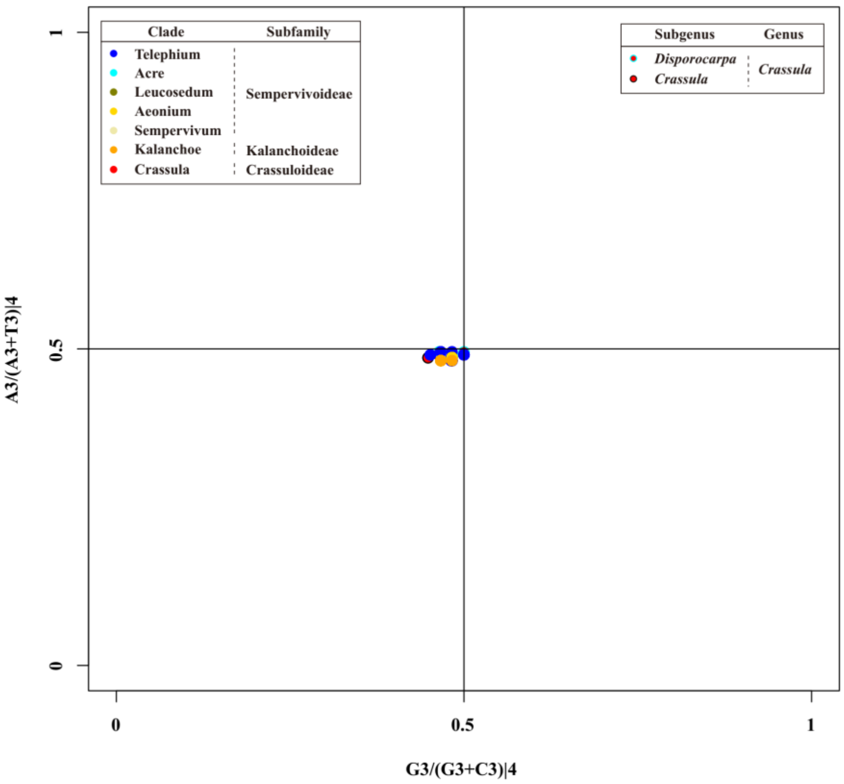
Clade
Subfamily
Telephium
Acre
Leucosedum
Aeonium
Sempervivum
Kalanchoe
Crassula
Sempervivoideae
Kalanchoideae
Crassuloideae
Subgenus
Genus
Disporocarpa
Crassula
Crassula
A3/(A3+T3)|4
G3/(G3+C3)|4
0
0.5
1
0
0.5
1

ndhC:

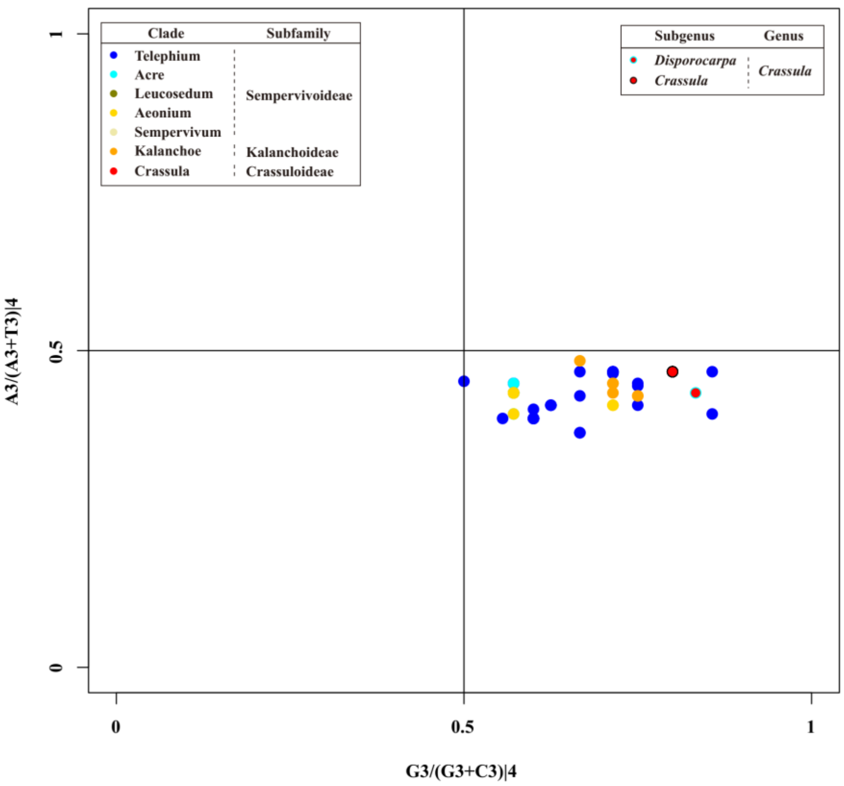
Clade
Subfamily
Telephium
Acre
Leucosedum
Aeonium
Sempervivum
Kalanchoe
Crassula
Sempervivoideae
Kalanchoideae
Crassuloideae
Subgenus
Genus
Disporocarpa
Crassula
Crassula
A3/(A3+T3)|4
G3/(G3+C3)|4
0
0.5
1
0
0.5
1

ndhD:

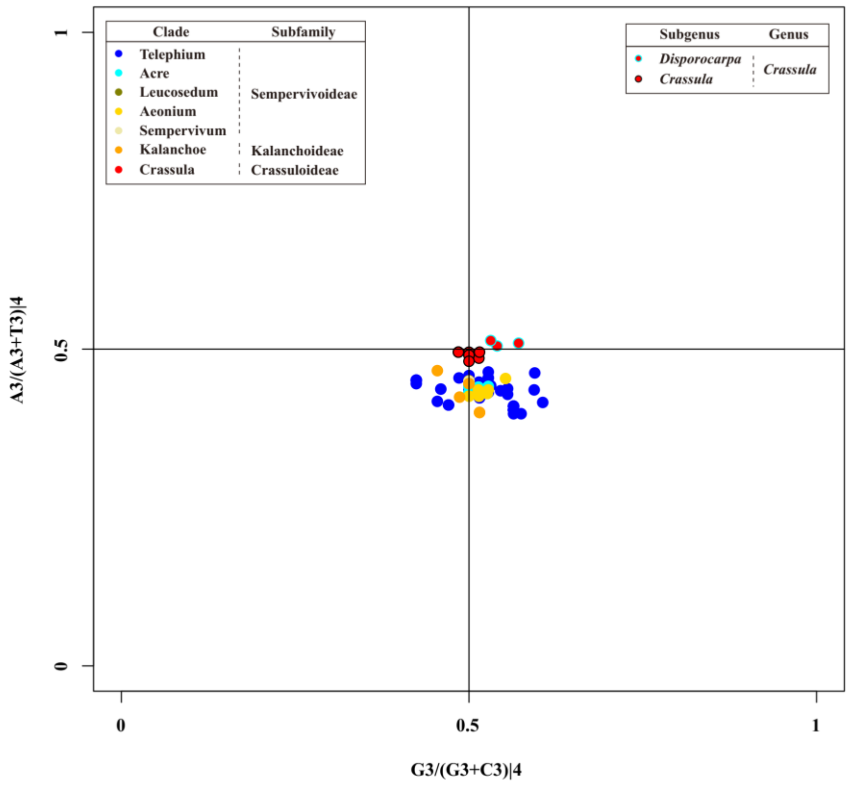
Clade
Subfamily
Telephium
Acre
Leucosedum
Aeonium
Sempervivum
Sempervivoideae
Kalanchoe
Kalanchoideae
Crassula
Crassuloideae
Subgenus
Genus
Disporocarpa
Crassula
Crassula
A3/(A3+T3)|4
G3/(G3+C3)|4
0
0.5
1

ndhE:

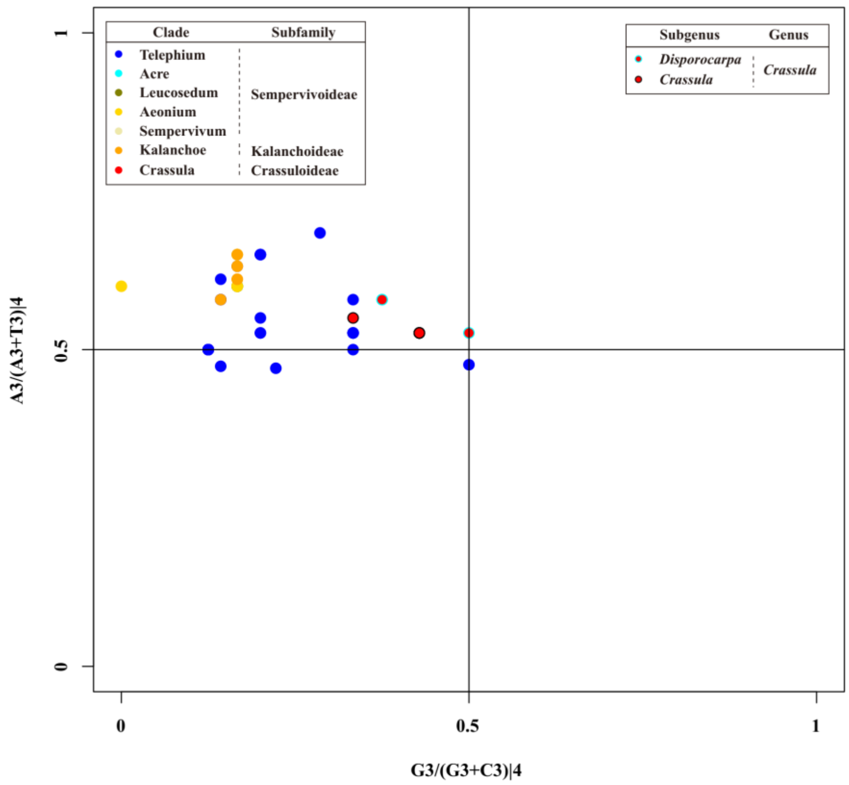
Clade
Subfamily
Telephium
Acre
Leucosedum
Aeonium
Sempervivum
Sempervivoideae
Kalanchoe
Kalanchoideae
Crassula
Crassuloideae
Subgenus
Genus
Disporocarpa
Crassula
Crassula
A3/(A3+T3)|4
G3/(G3+C3)|4
0
0.5
1

ndhF:

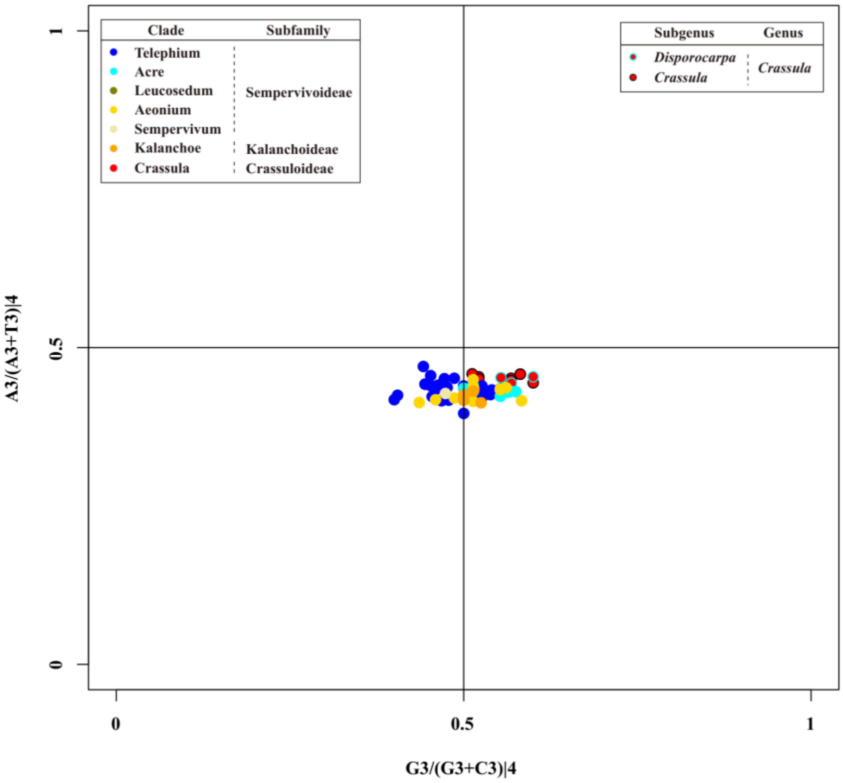
Clade
Subfamily
Telephium
Acre
Leucosedum
Aeonium
Sempervivum
Kalanchoe
Crassula
Sempervivoideae
Kalanchoideae
Crassuloideae
Subgenus
Genus
Disporocarpa
Crassula
Crassula
1
0.5
0
0
0.5
1
A3/(A3+T3)|4
G3/(G3+C3)|4

ndhG:

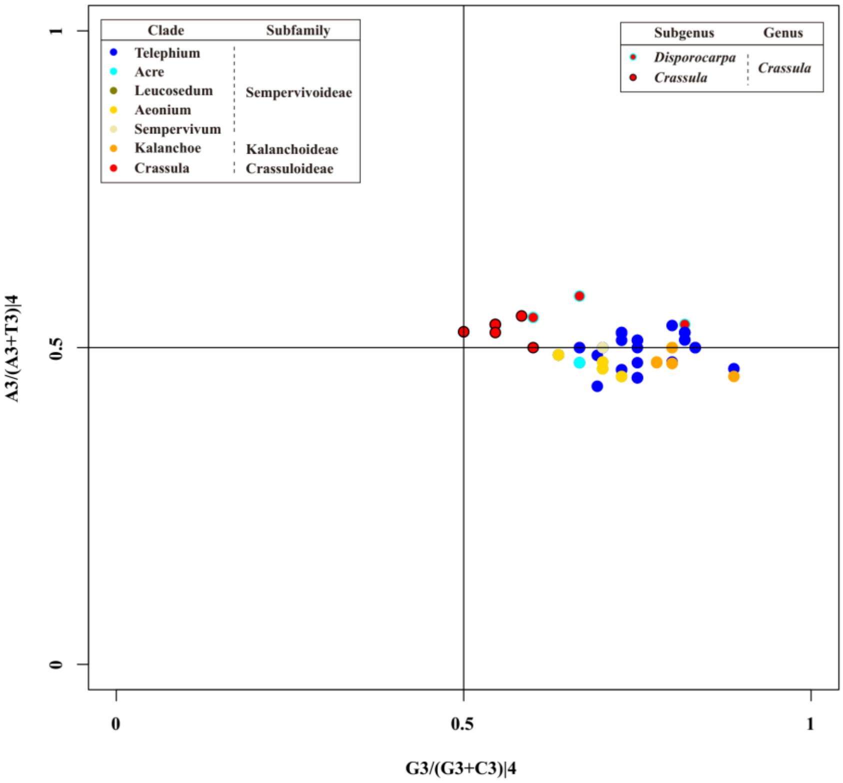
Clade
Subfamily
Telephium
Acre
Leucosedum
Aeonium
Sempervivum
Kalanchoe
Crassula
Sempervivoideae
Kalanchoideae
Crassuloideae
Subgenus
Genus
Disporocarpa
Crassula
Crassula
1
0.5
0
0
0.5
1
A3/(A3+T3)|4
G3/(G3+C3)|4

ndhH:

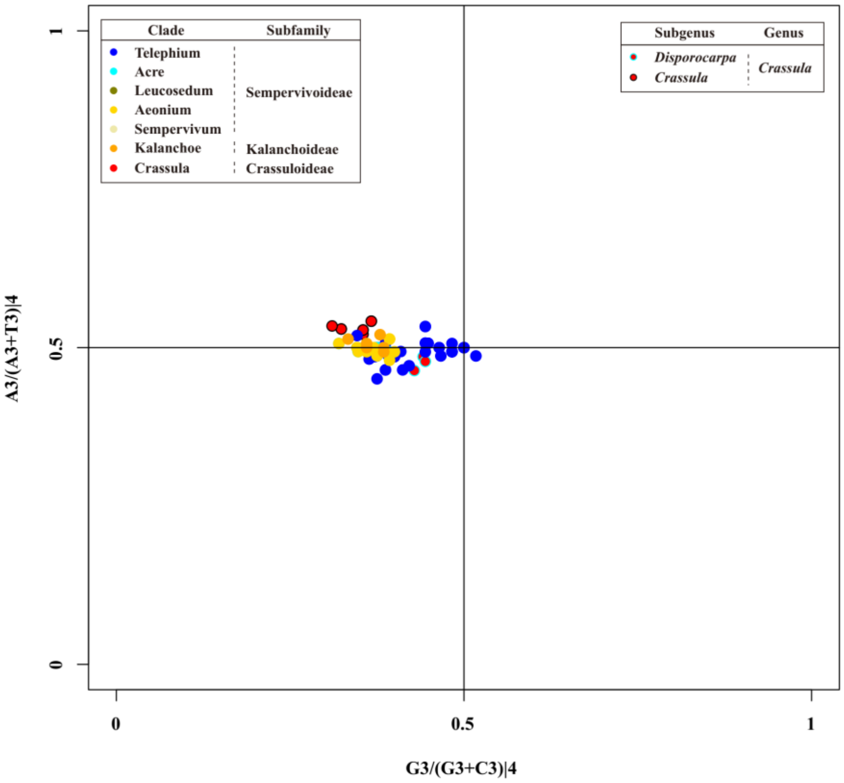

Clade
Subfamily
Telephium
Acre
Leucosedum
Aeonium
Sempervivum
Kalanchoe
Crassula
Sempervivoideae
Kalanchoideae
Crassuloideae
Subgenus
Genus
Disporocarpa
Crassula
Crassula
A3/(A3+T3)|4
G3/(G3+C3)|4
0
0.5
1

ndhI:

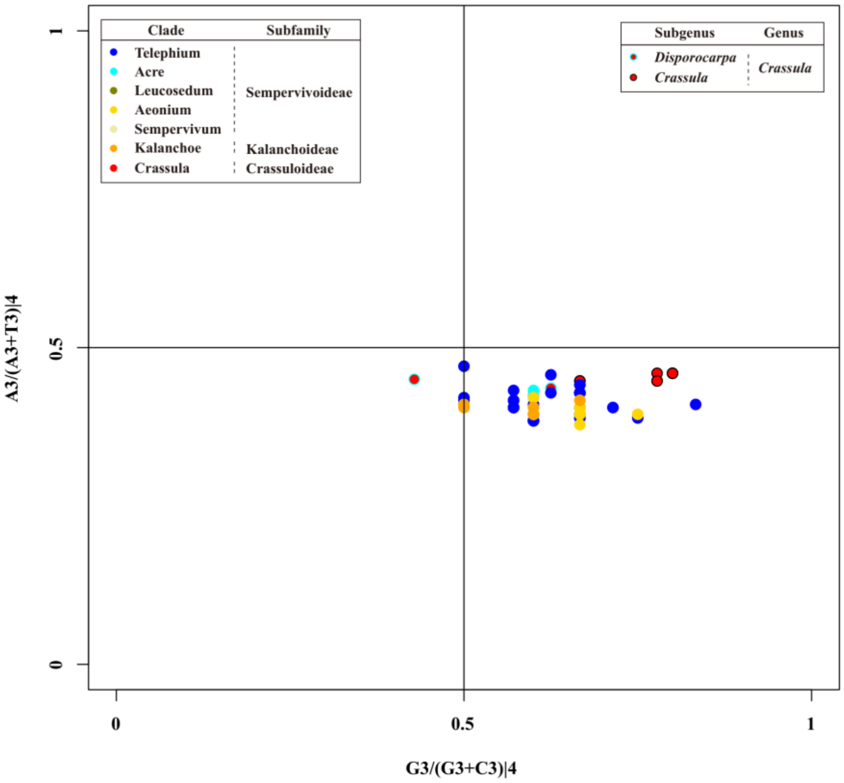

Clade
Subfamily
Telephium
Acre
Leucosedum
Aeonium
Sempervivum
Kalanchoe
Crassula
Sempervivoideae
Kalanchoideae
Crassuloideae
Subgenus
Genus
Disporocarpa
Crassula
Crassula
A3/(A3+T3)|4
G3/(G3+C3)|4
0
0.5
1

ndhJ:

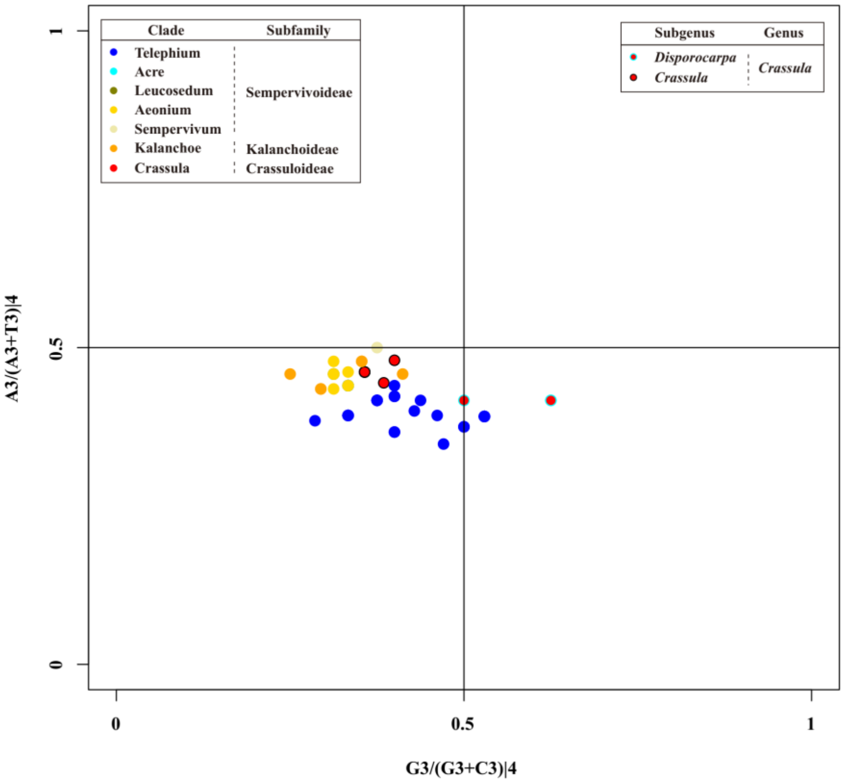
Clade
Subfamily
Telephium
Acre
Leucosedum
Aeonium
Sempervivum
Kalanchoe
Crassula
Sempervivoideae
Kalanchoideae
Crassuloideae
Subgenus
Genus
Disporocarpa
Crassula
Crassula
A3/(A3+T3)|4
G3/(G3+C3)|4
0
0.5
1

ndhK:

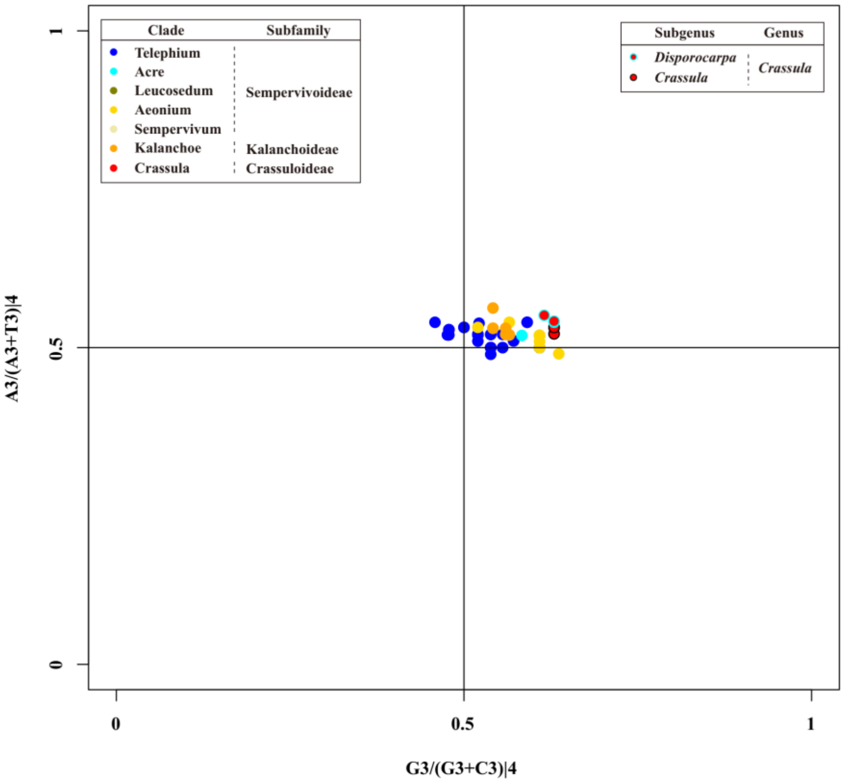
Clade
Subfamily
Telephium
Acre
Leucosedum
Aeonium
Sempervivum
Kalanchoe
Crassula
Sempervivoideae
Kalanchoideae
Crassuloideae
Subgenus
Genus
Disporocarpa
Crassula
Crassula
A3/(A3+T3)|4
G3/(G3+C3)|4
0
0.5
1

pafI:

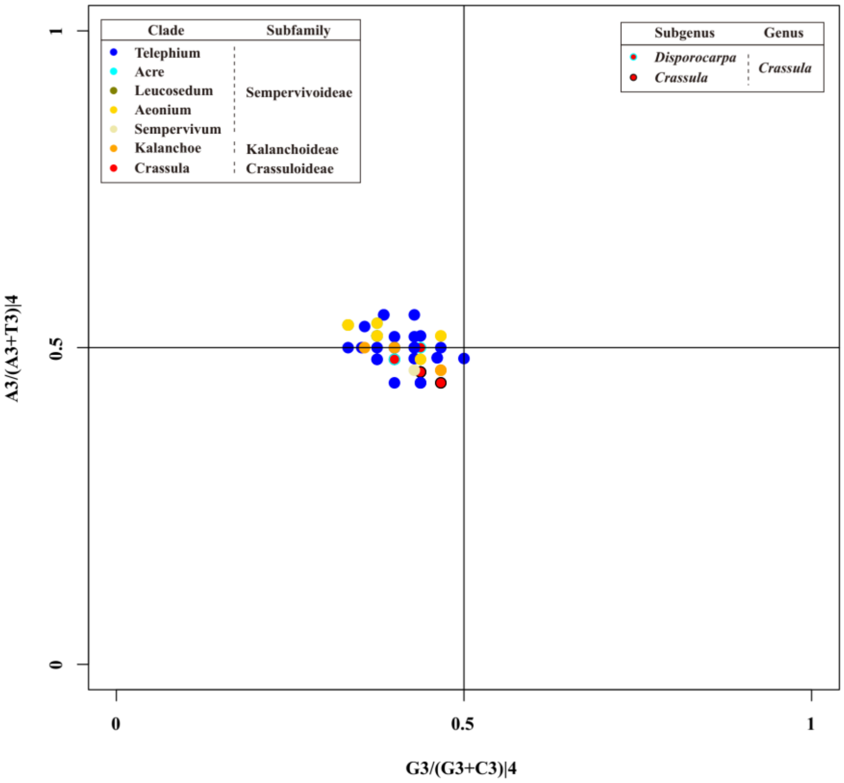
Clade
Subfamily
Telephium
Acre
Leucosedum
Aeonium
Sempervivum
Kalanchoe
Crassula
Sempervivoideae
Kalanchoideae
Crassuloideae
Subgenus
Genus
Disporocarpa
Crassula
Crassula
A3/(A3+T3)|4
G3/(G3+C3)|4
0
0.5
1

pafII:

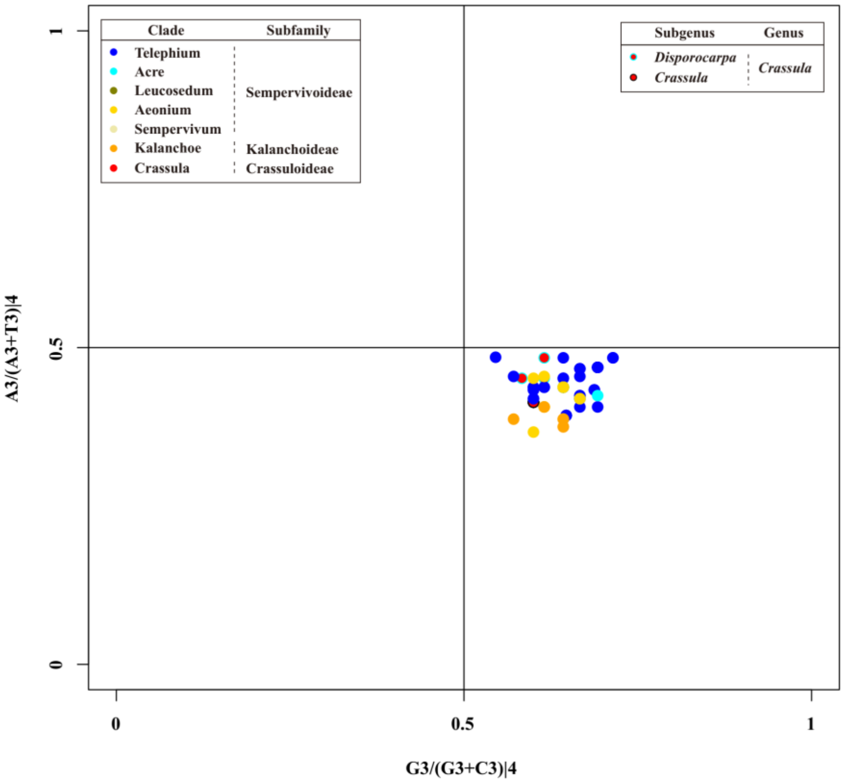
Clade
Subfamily
Telephium
Acre
Leucosedum
Aeonium
Sempervivum
Kalanchoe
Crassula
Sempervivoideae
Kalanchoideae
Crassuloideae
Subgenus
Genus
Disporocarpa
Crassula
Crassula
A3/(A3+T3)|4
G3/(G3+C3)|4
0
0.5
1

petA:

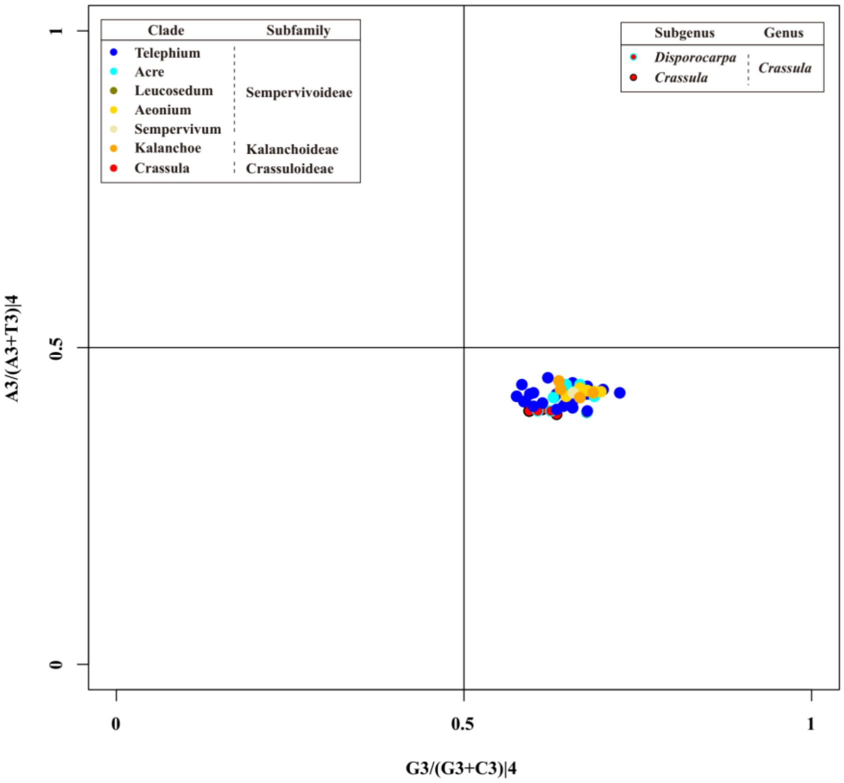
Clade
Subfamily
Telephium
Acre
Leucosedum
Aeonium
Sempervivum
Sempervivoideae
Kalanchoe
Kalanchoideae
Crassula
Crassuloideae
Subgenus
Genus
Disporocarpa
Crassula
Crassula
A3/(A3+T3)|4
G3/(G3+C3)|4
0
0.5
1
0
0.5
1

petB:

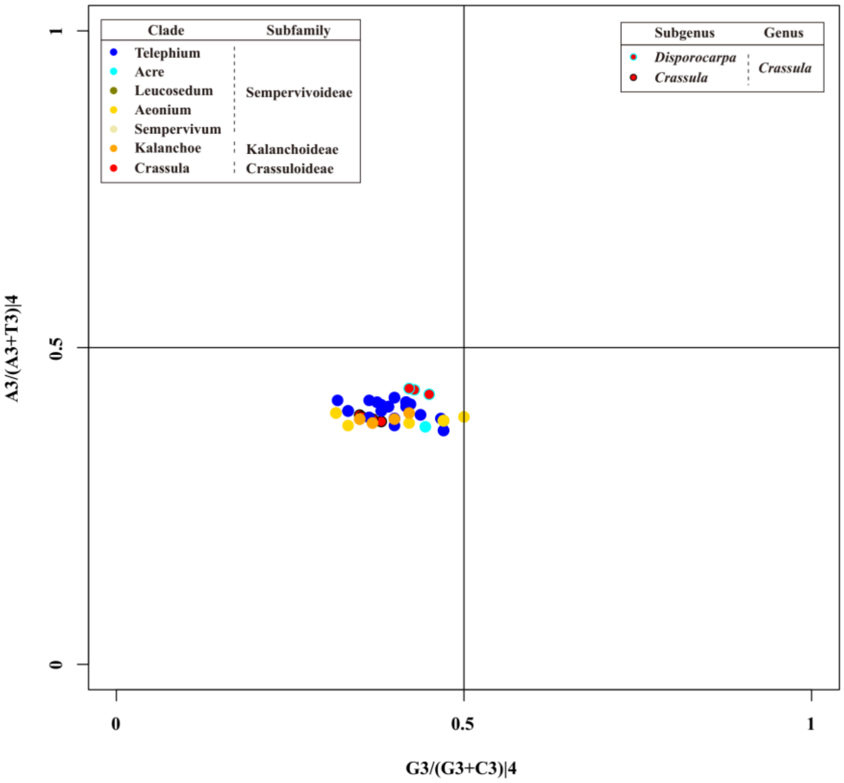
Clade
Subfamily
Telephium
Acre
Leucosedum
Aeonium
Sempervivum
Sempervivoideae
Kalanchoe
Kalanchoideae
Crassula
Crassuloideae
Subgenus
Genus
Disporocarpa
Crassula
Crassula
A3/(A3+T3)|4
G3/(G3+C3)|4
0
0.5
1
0
0.5
1

petD:

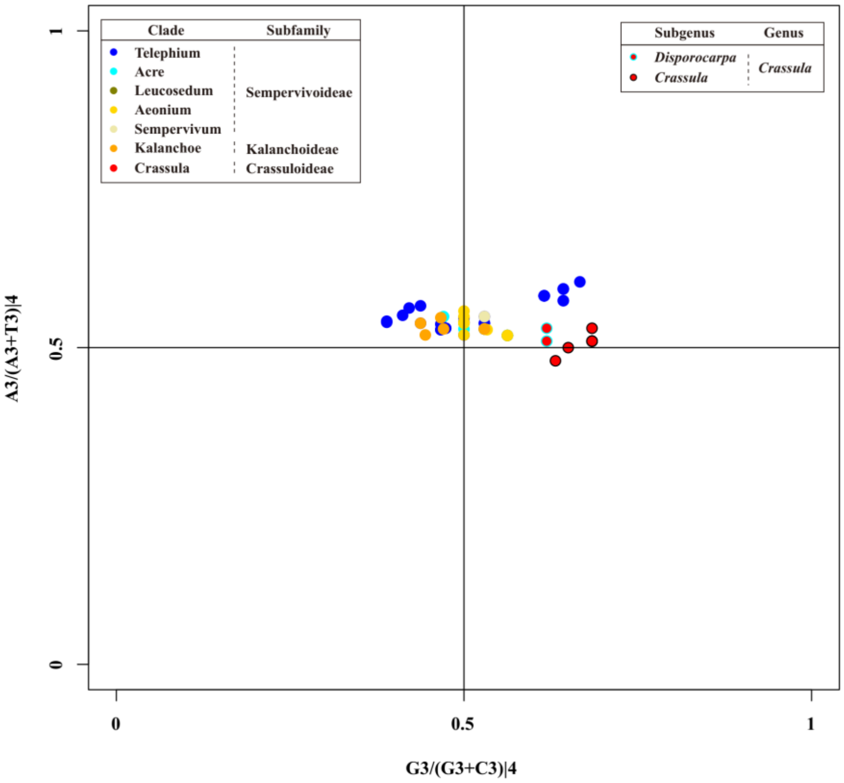

Clade
Subfamily
Telephium
Acre
Leucosedum
Aeonium
Sempervivum
Kalanchoe
Crassula
Sempervivoideae
Kalanchoideae
Crassuloideae
Subgenus
Genus
Disporocarpa
Crassula
Crassula
A3/(A3+T3)|4
G3/(G3+C3)|4
0
0.5
1

psaA:

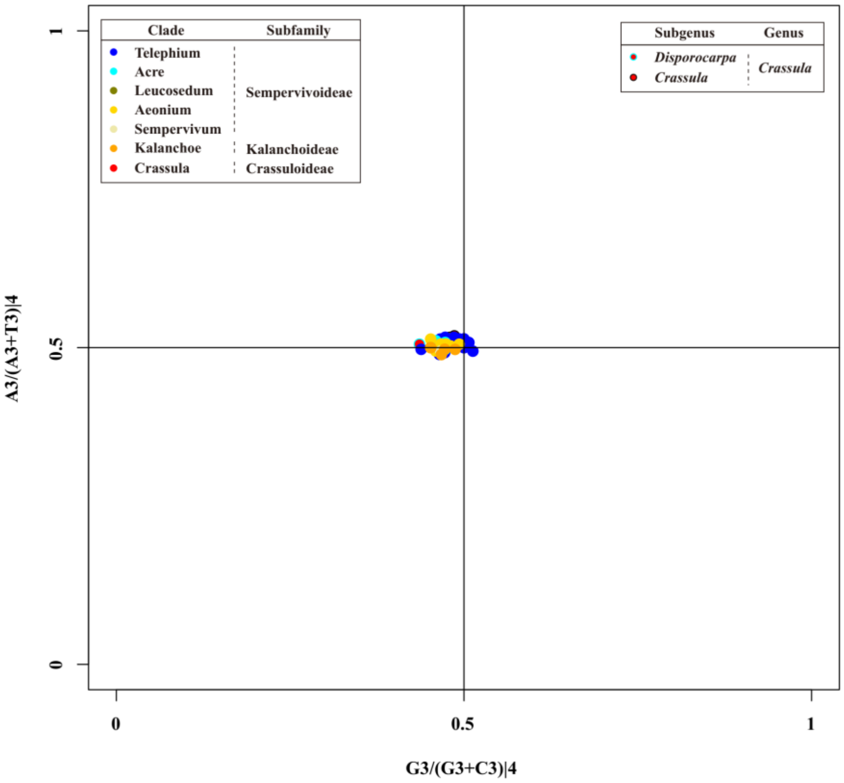

Clade
Subfamily
Telephium
Acre
Leucosedum
Aeonium
Sempervivum
Kalanchoe
Crassula
Sempervivoideae
Kalanchoideae
Crassuloideae
Subgenus
Genus
Disporocarpa
Crassula
Crassula
A3/(A3+T3)|4
G3/(G3+C3)|4
0
0.5
1

psaB:

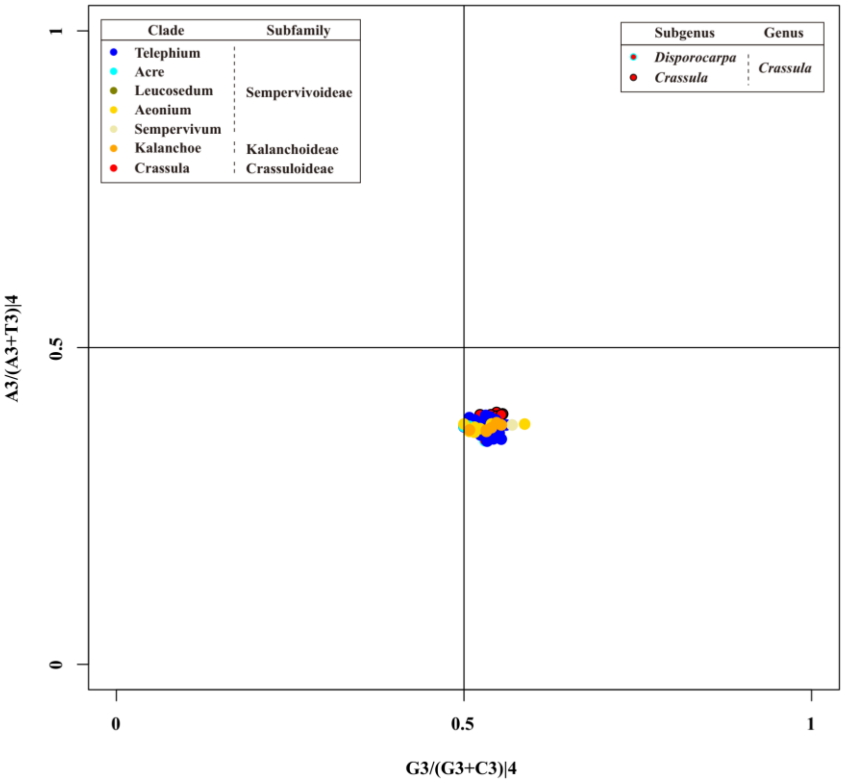

Clade
Subfamily
Telephium
Acre
Leucosedum
Aeonium
Sempervivum
Kalanchoe
Crassula
Sempervivoideae
Kalanchoideae
Crassuloideae
Subgenus
Genus
Disporocarpa
Crassula
Crassula
A3/(A3+T3)|4
G3/(G3+C3)|4
0
0.5
1

psbA:

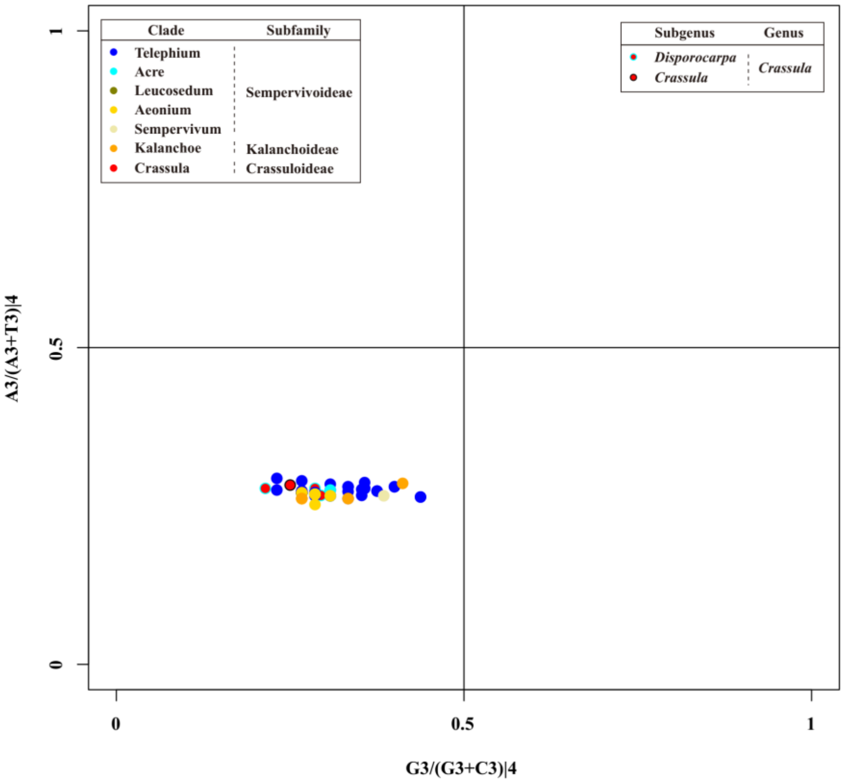

Clade
Subfamily
Telephium
Acre
Leucosedum
Aeonium
Sempervivum
Kalanchoe
Crassula
Sempervivoideae
Kalanchoideae
Crassuloideae
Subgenus
Genus
Disporocarpa
Crassula
Crassula
A3/(A3+T3)|4
G3/(G3+C3)|4
0
0.5
1

psbB:

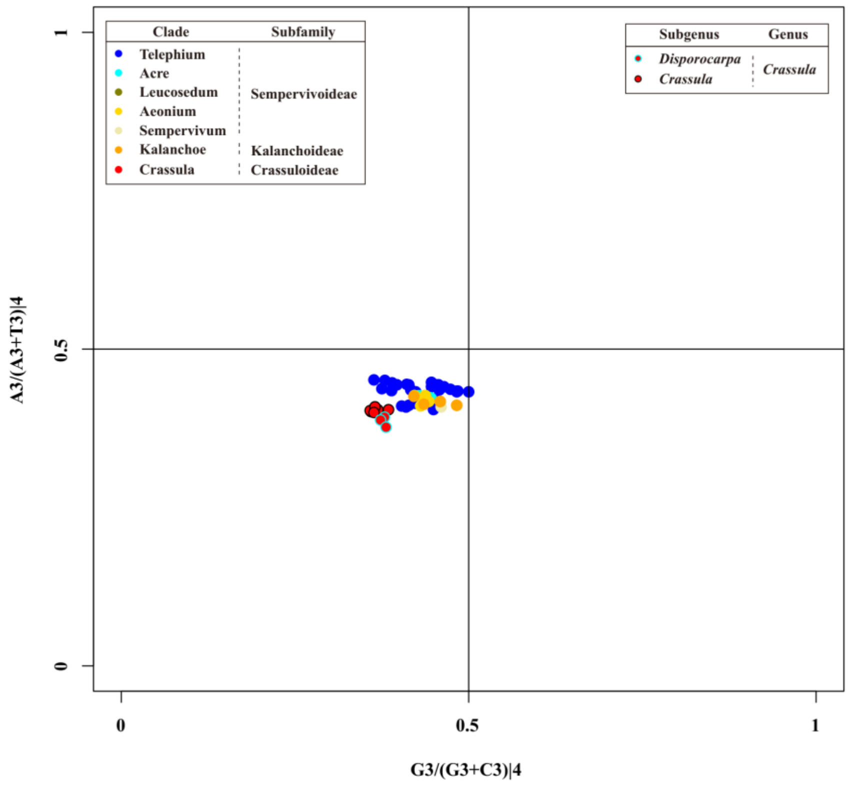

Clade
Subfamily
Telephium
Acre
Leucosedum
Aeonium
Sempervivum
Kalanchoe
Crassula
Sempervivoideae
Kalanchoideae
Crassuloideae
Subgenus
Genus
Disporocarpa
Crassula
Crassula
1
0.5
0
A3/(A3+T3)|4
0
0.5
1
G3/(G3+C3)|4

psbC:

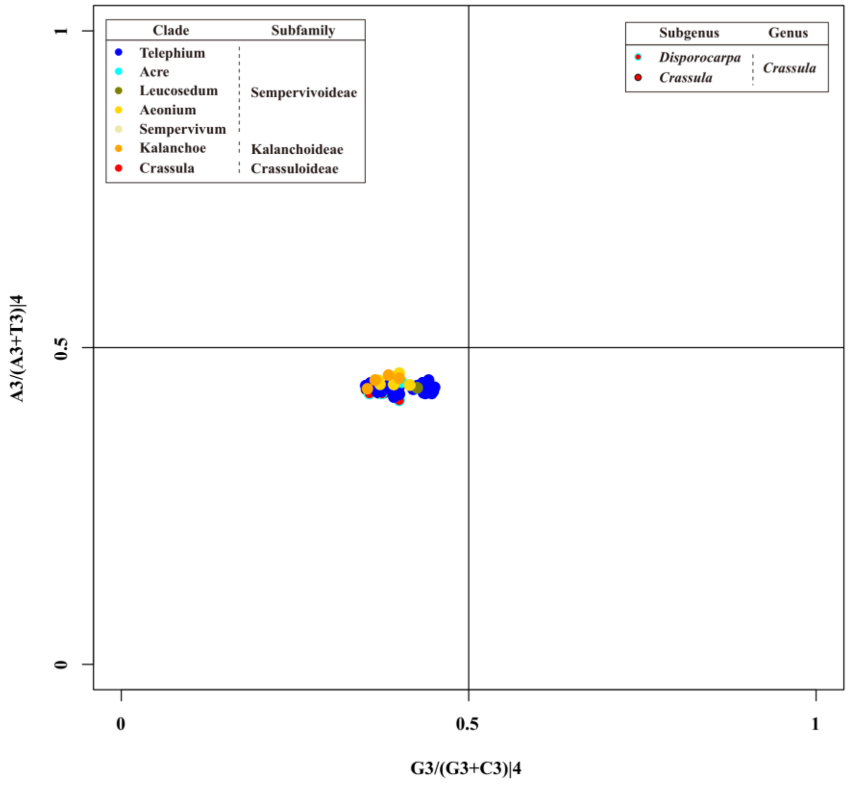

Clade
Subfamily
Telephium
Acre
Leucosedum
Aeonium
Sempervivum
Kalanchoe
Crassula
Sempervivoideae
Kalanchoideae
Crassuloideae
Subgenus
Genus
Disporocarpa
Crassula
Crassula
1
0.5
0
A3/(A3+T3)|4
0
0.5
1
G3/(G3+C3)|4

psbD:

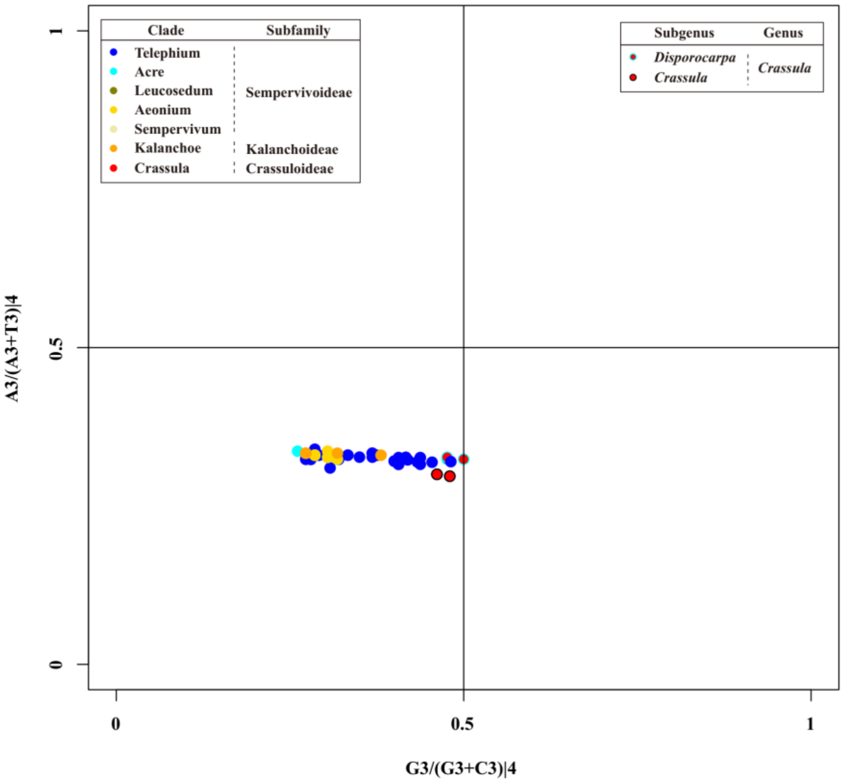

Clade
Subfamily
Telephium
Acre
Leucosedum
Aeonium
Sempervivum
Sempervivoideae
Kalanchoe
Kalanchoideae
Crassula
Crassuloideae
Subgenus
Genus
Disporocarpa
Crassula
Crassula
1
0.5
0
A3/(A3+T3)|4
0
0.5
1
G3/(G3+C3)|4

rbcL:

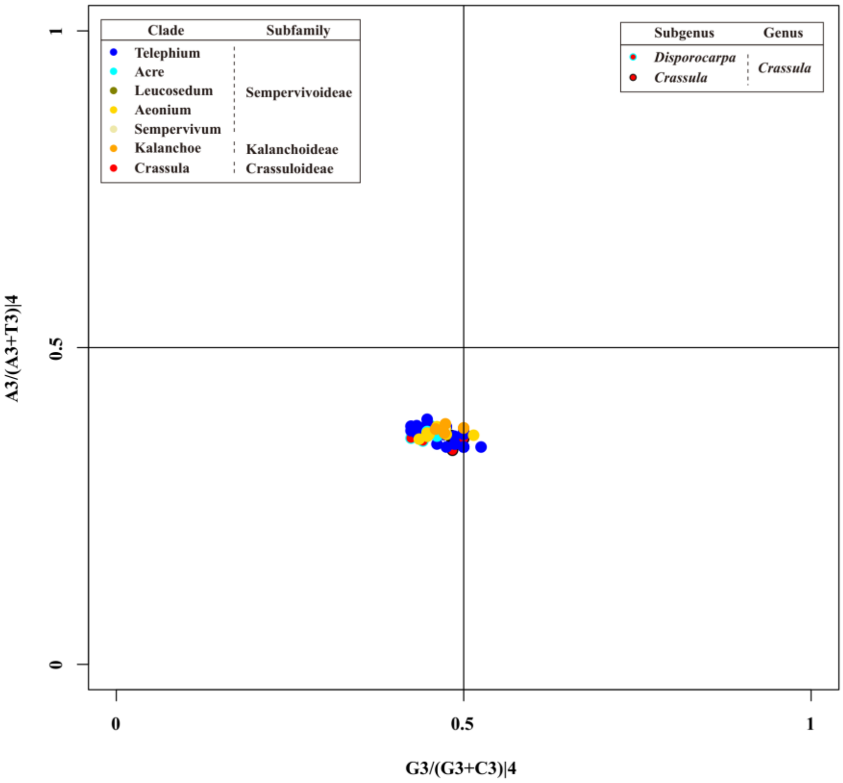

Clade
Subfamily
Telephium
Acre
Leucosedum
Aeonium
Sempervivum
Sempervivoideae
Kalanchoe
Kalanchoideae
Crassula
Crassuloideae
Subgenus
Genus
Disporocarpa
Crassula
Crassula
1
0.5
0
A3/(A3+T3)|4
0
0.5
1
G3/(G3+C3)|4

rpl2:

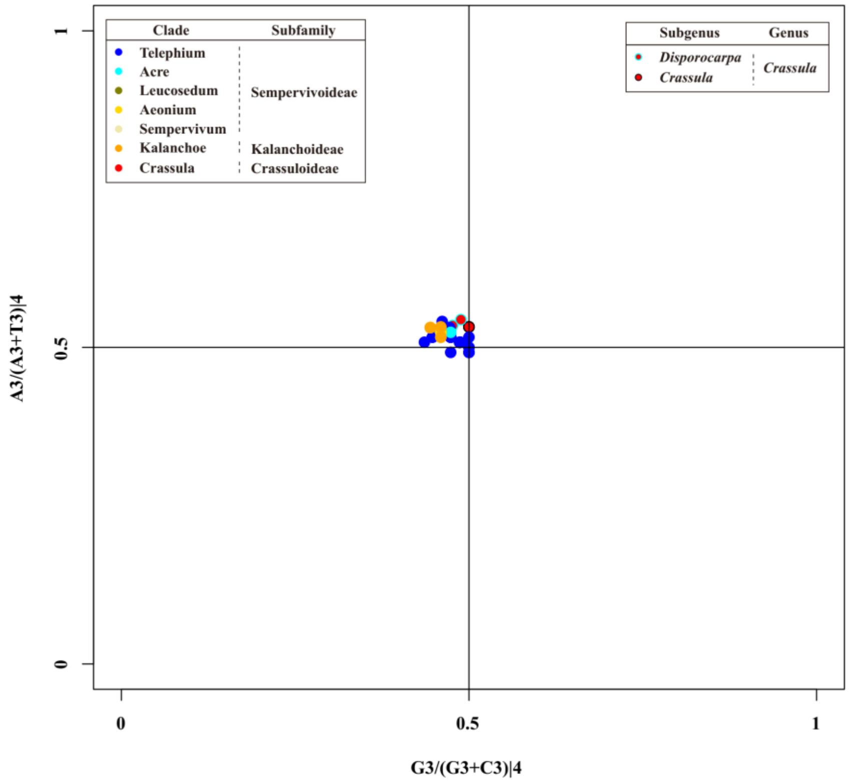
Clade
Subfamily
Telephium
Acre
Leucosedum
Aeonium
Sempervivum
Kalanchoe
Crassula
Sempervivoideae
Kalanchoideae
Crassuloideae
Subgenus
Genus
Disporocarpa
Crassula
Crassula
A3/(A3+T3)|4
G3/(G3+C3)|4
0
0.5
1
0
0.5
1

rpl14:

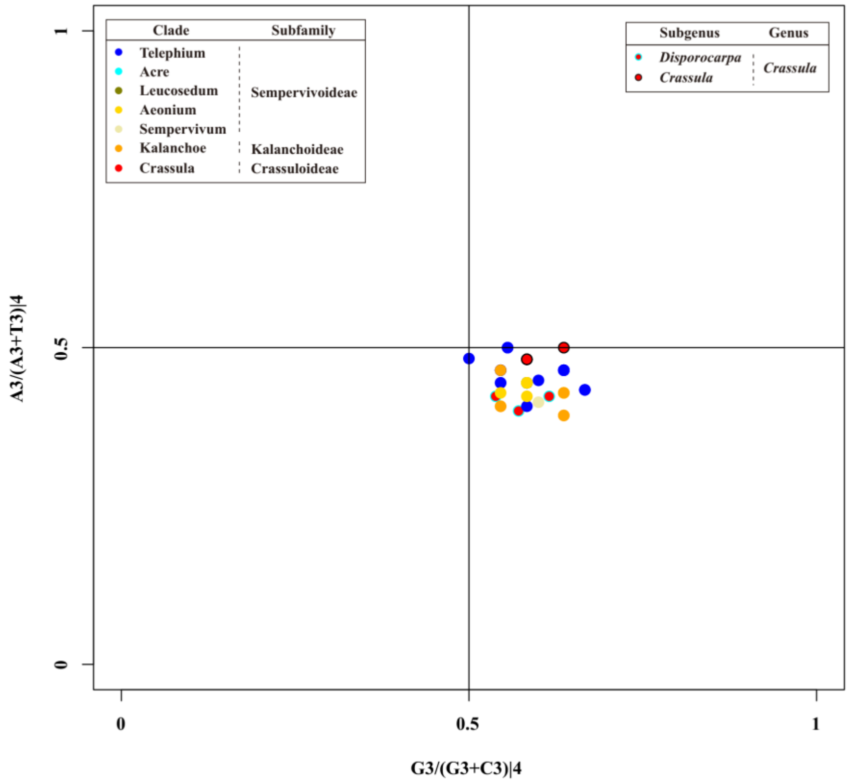
Clade
Subfamily
Telephium
Acre
Leucosedum
Aeonium
Sempervivum
Kalanchoe
Crassula
Sempervivoideae
Kalanchoideae
Crassuloideae
Subgenus
Genus
Disporocarpa
Crassula
Crassula
A3/(A3+T3)|4
G3/(G3+C3)|4
0
0.5
1
0
0.5
1

rpl16:

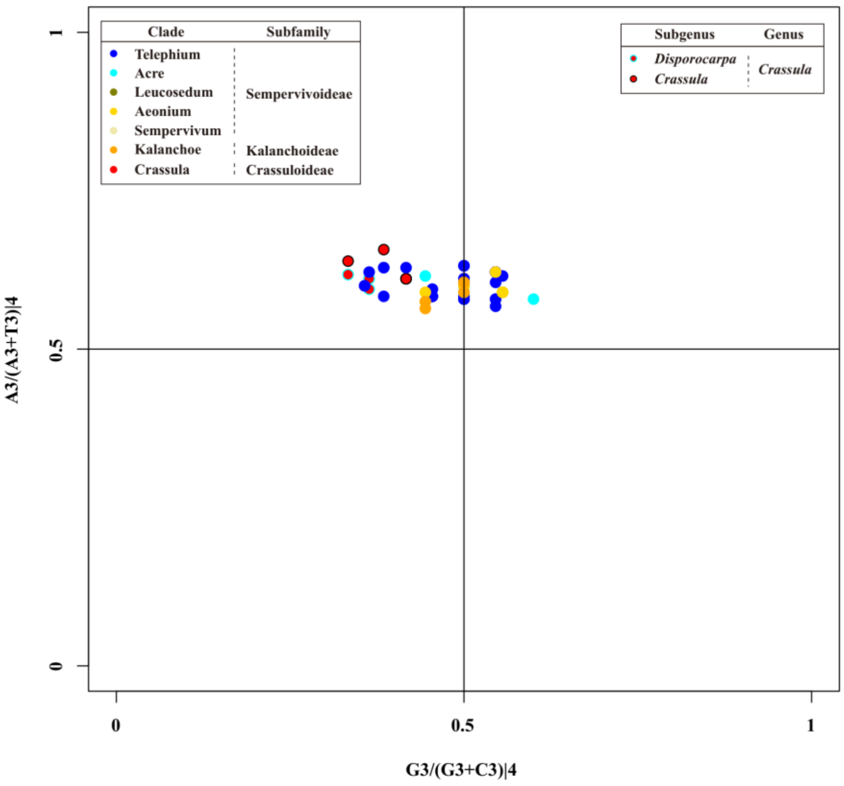
Clade
Subfamily
Telephium
Acre
Leucosedum
Aeonium
Sempervivum
Kalanchoe
Crassula
Sempervivoideae
Kalanchoideae
Crassuloideae
Subgenus
Genus
Disporocarpa
Crassula
Crassula
A3/(A3+T3)|4
G3/(G3+C3)|4
0
0.5
1
0
0.5
1

rpl20:

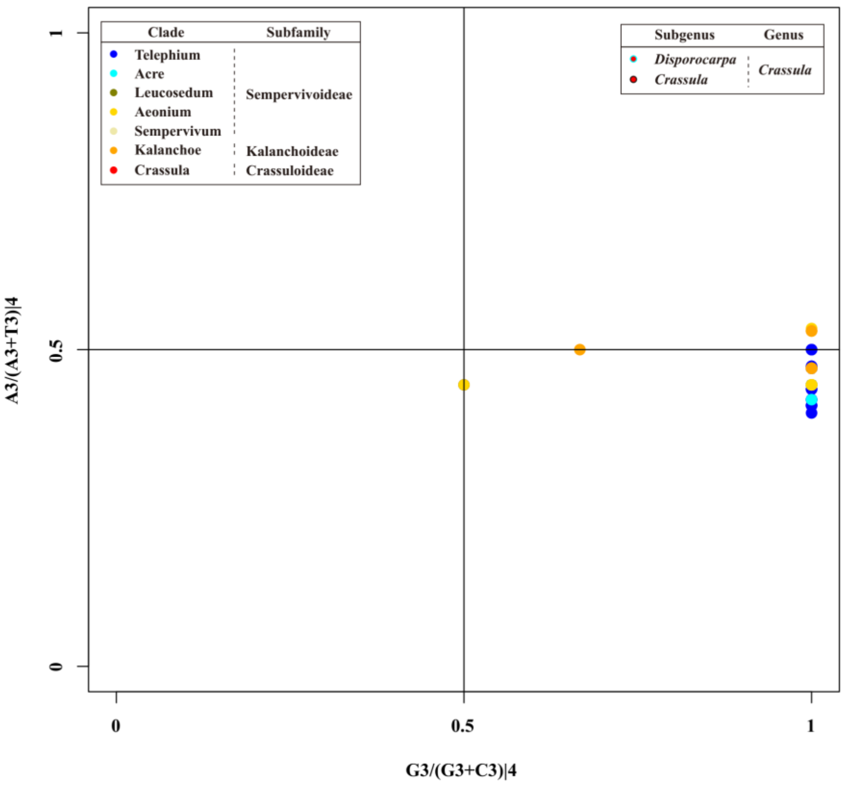
Clade
Subfamily
Telephium
Acre
Leucosedum
Aeonium
Sempervivum
Kalanchoe
Crassula
Sempervivoideae
Kalanchoideae
Crassuloideae
Subgenus
Genus
Disporocarpa
Crassula
Crassula
A3/(A3+T3)|4
G3/(G3+C3)|4
0
0.5
1
0
0.5
1

rpl22:

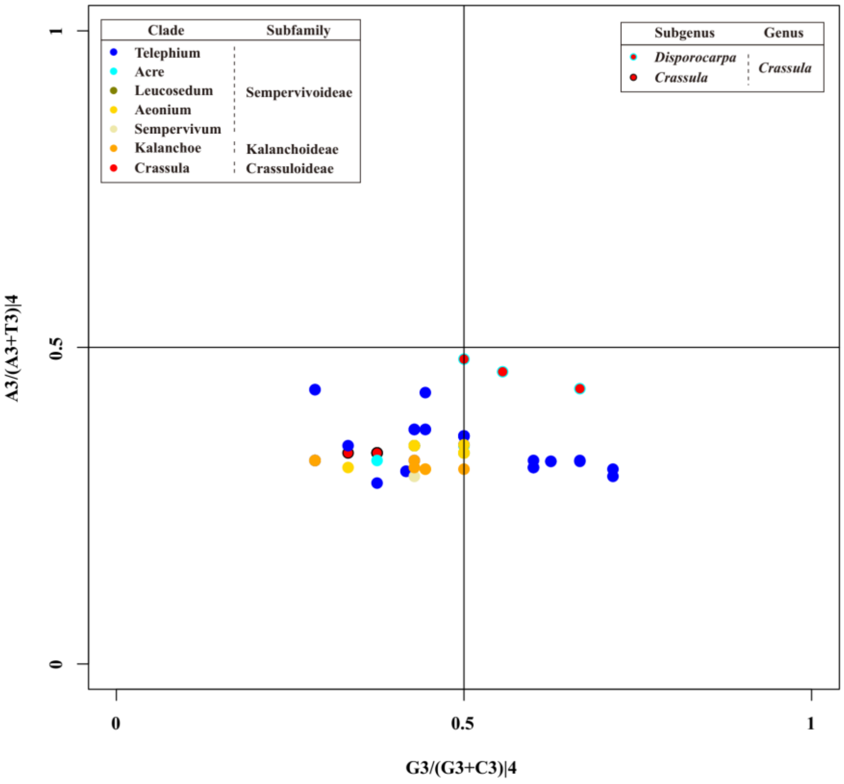
Clade
Subfamily
Telephium
Acre
Leucosedum
Aeonium
Sempervivum
Kalanchoe
Crassula
Sempervivoideae
Kalanchoideae
Crassuloideae
Subgenus
Genus
Disporocarpa
Crassula
Crassula
A3/(A3+T3)|4
G3/(G3+C3)|4
0
0.5
1

rpoA:

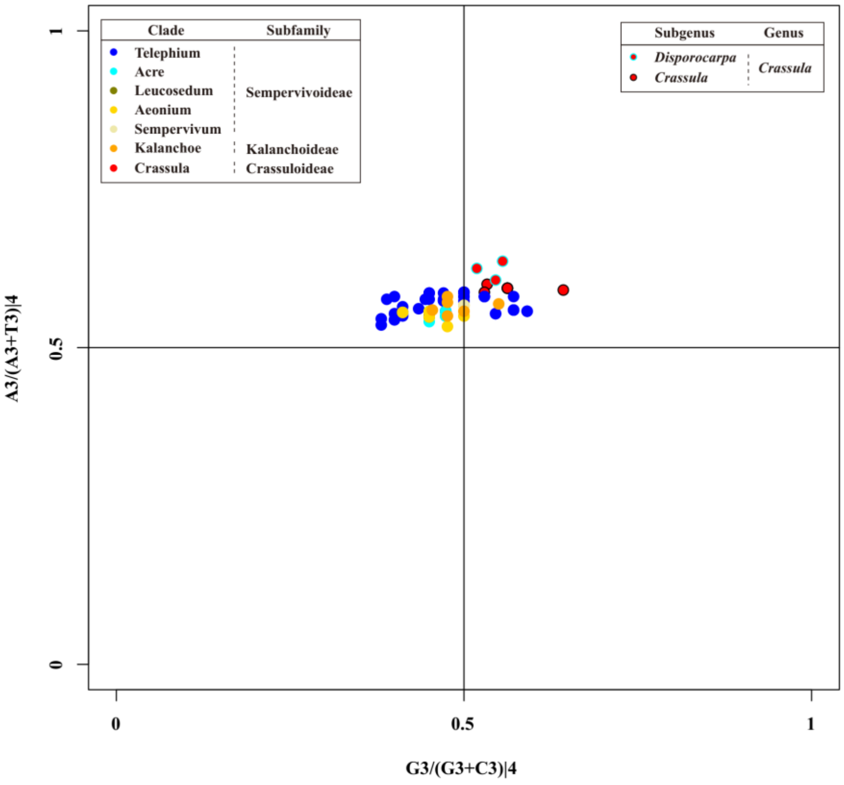
Clade
Subfamily
Telephium
Acre
Leucosedum
Aeonium
Sempervivum
Kalanchoe
Crassula
Sempervivoideae
Kalanchoideae
Crassuloideae
Subgenus
Genus
Disporocarpa
Crassula
Crassula
A3/(A3+T3)|4
G3/(G3+C3)|4
0
0.5
1

rpoB:

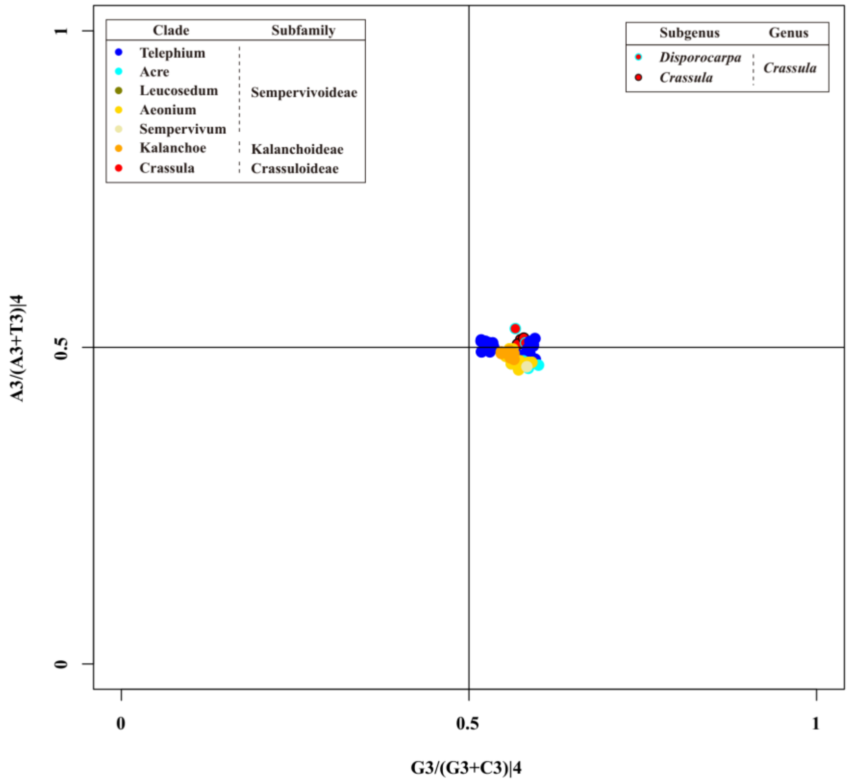
Clade
Subfamily
Telephium
Acre
Leucosedum
Aeonium
Sempervivum
Sempervivoideae
Kalanchoe
Kalanchoideae
Crassula
Crassuloideae
Subgenus
Genus
Disporocarpa
Crassula
Crassula
A3/(A3+T3)|4
G3/(G3+C3)|4
0
0.5
1

rpoC1:

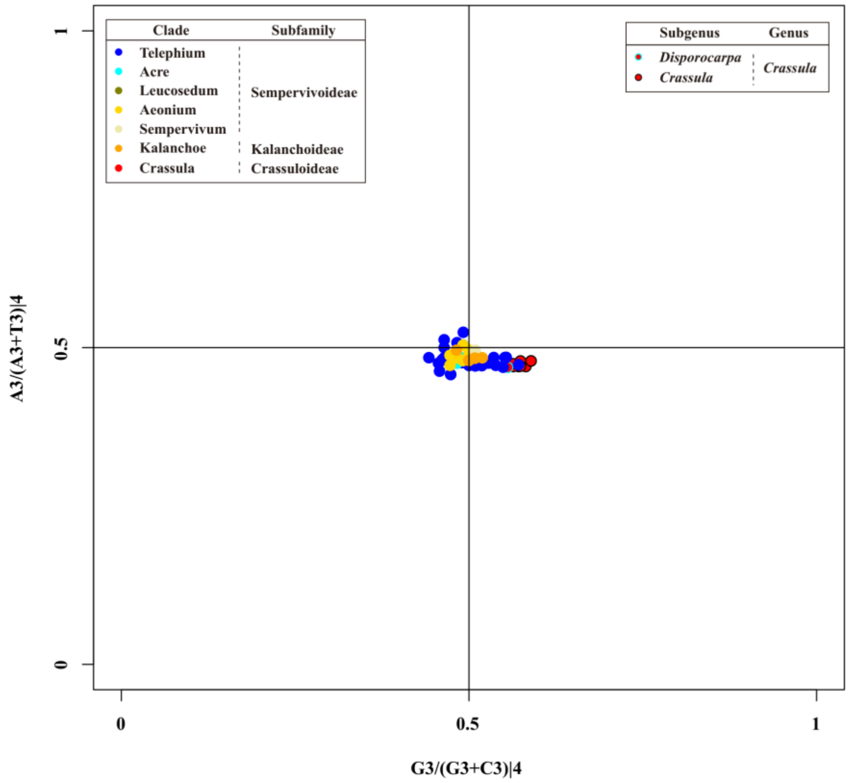
Clade
Subfamily
Telephium
Acre
Leucosedum
Aeonium
Sempervivum
Sempervivoideae
Kalanchoe
Kalanchoideae
Crassula
Crassuloideae
Subgenus
Genus
Disporocarpa
Crassula
Crassula
A3/(A3+T3)|4
G3/(G3+C3)|4
0
0.5
1

rpoC2:

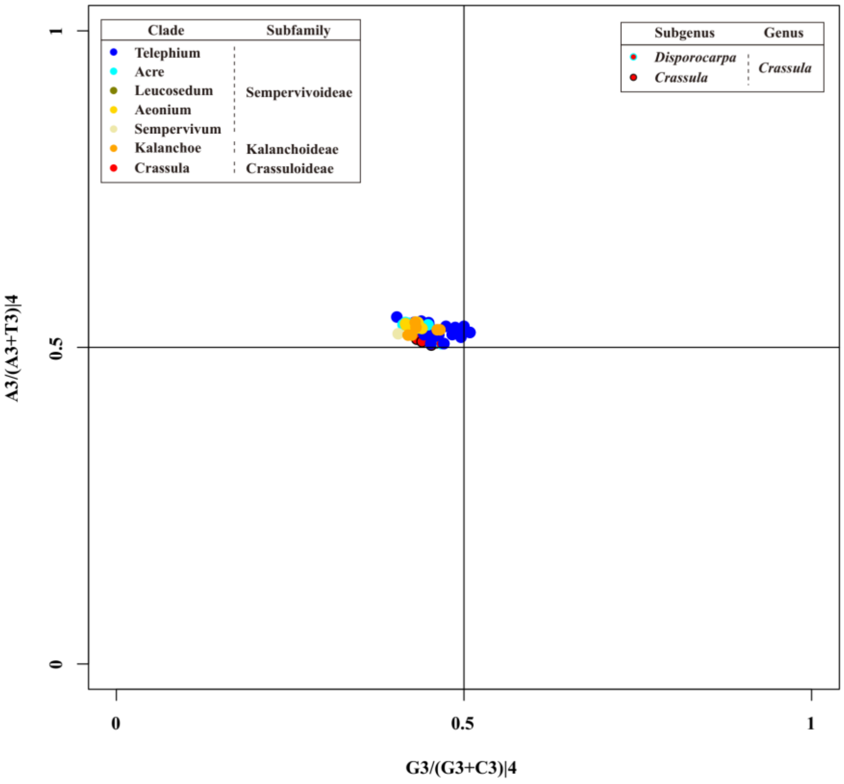

Clade
Subfamily
Telephium
Acre
Leucosedum
Aeonium
Sempervivum
Sempervivoideae
Kalanchoe
Kalanchoideae
Crassula
Crassuloideae
Subgenus
Genus
Disporocarpa
Crassula
Crassula
A3/(A3+T3)|4
G3/(G3+C3)|4
0
0.5
1

rps2:

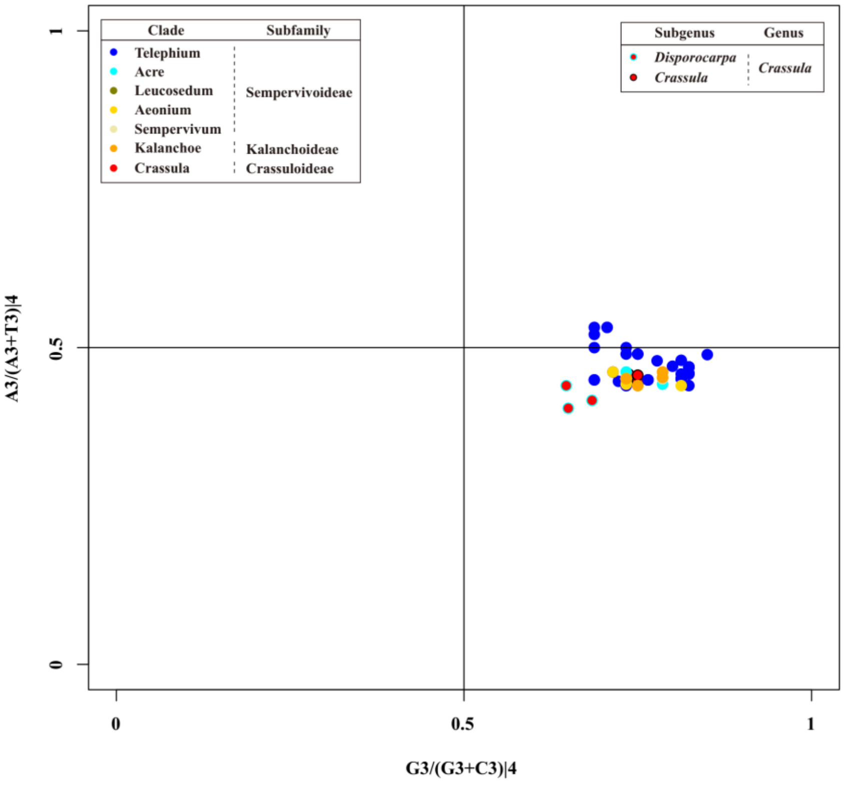

Clade
Subfamily
Telephium
Acre
Leucosedum
Aeonium
Sempervivum
Sempervivoideae
Kalanchoe
Kalanchoideae
Crassula
Crassuloideae
Subgenus
Genus
Disporocarpa
Crassula
Crassula
A3/(A3+T3)|4
G3/(G3+C3)|4
0
0.5
1

rps3:

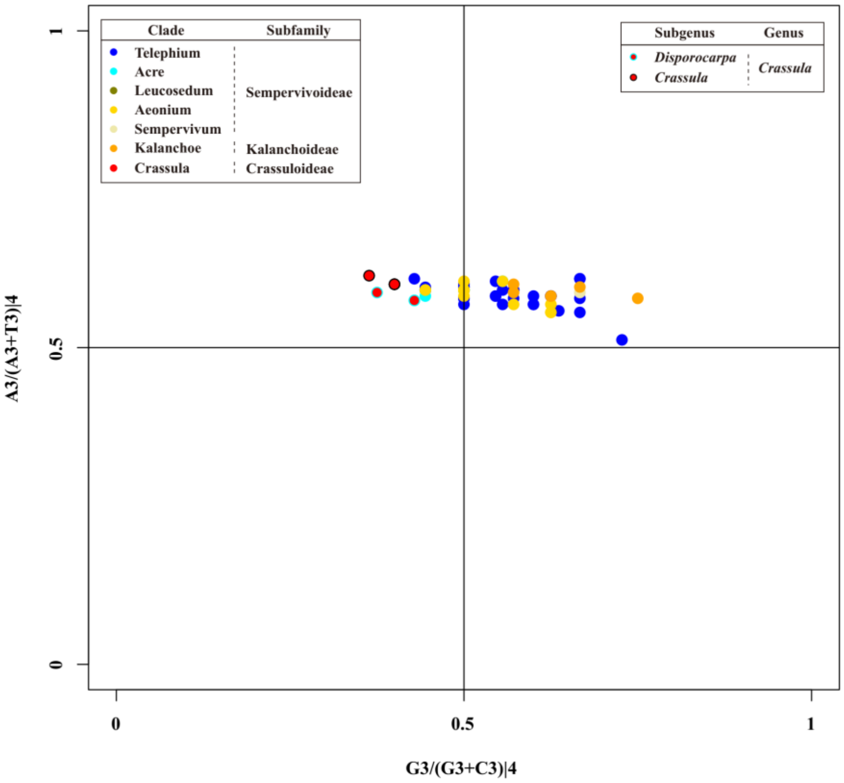
Clade
Subfamily
Telephium
Acre
Leucosedum
Aeonium
Sempervivum
Kalanchoe
Crassula
Sempervivoideae
Kalanchoideae
Crassuloideae
Subgenus
Genus
Disporocarpa
Crassula
Crassula
A3/(A3+T3)|4
G3/(G3+C3)|4
0
0.5
1
0
0.5
1

rps4:

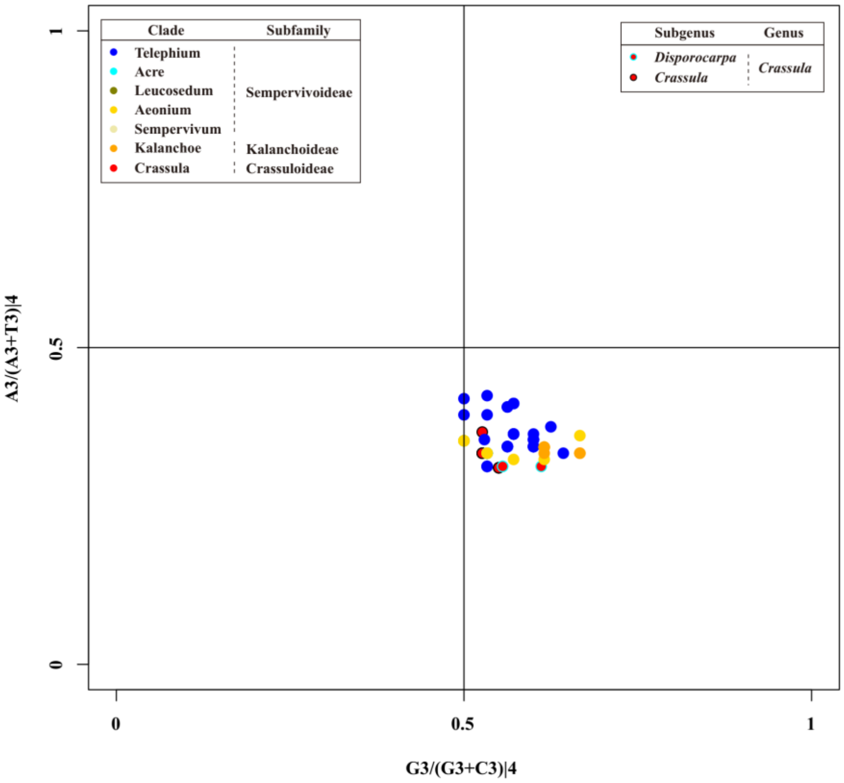
Clade
Subfamily
Telephium
Acre
Leucosedum
Aeonium
Sempervivum
Kalanchoe
Crassula
Sempervivoideae
Kalanchoideae
Crassuloideae
Subgenus
Genus
Disporocarpa
Crassula
Crassula
A3/(A3+T3)|4
G3/(G3+C3)|4
0
0.5
1
0
0.5
1

rps7:

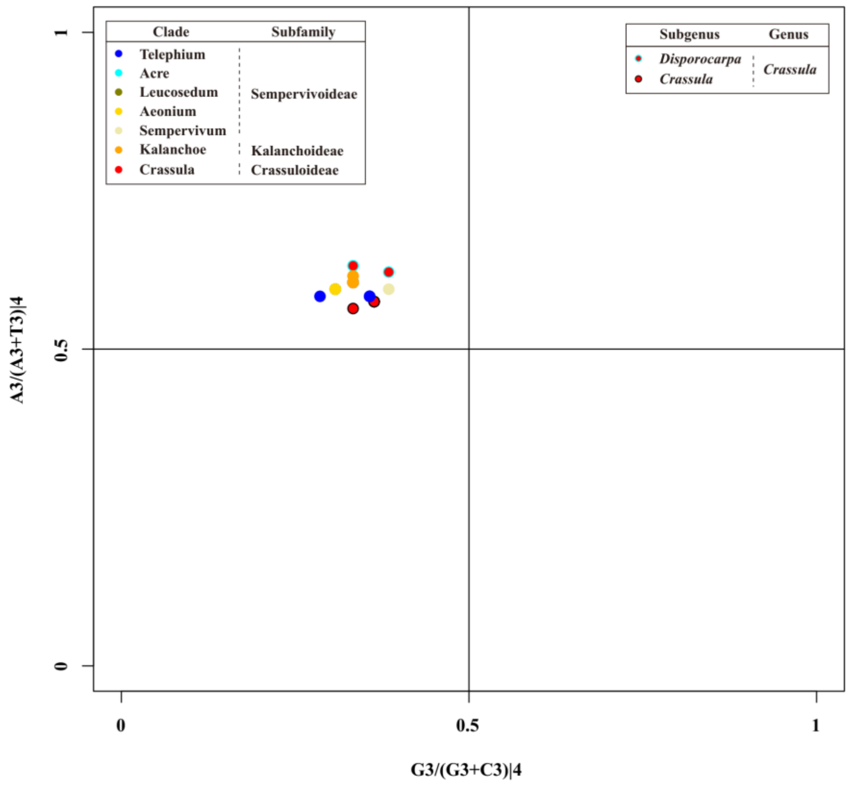
Clade
Subfamily
Telephium
Acre
Leucosedum
Aeonium
Sempervivum
Kalanchoe
Crassula
Sempervivoideae
Kalanchoideae
Crassuloideae
Subgenus
Genus
Disporocarpa
Crassula
Crassula
A3/(A3+T3)|4
G3/(G3+C3)|4
0
0.5
1
0
0.5
1

rps8:

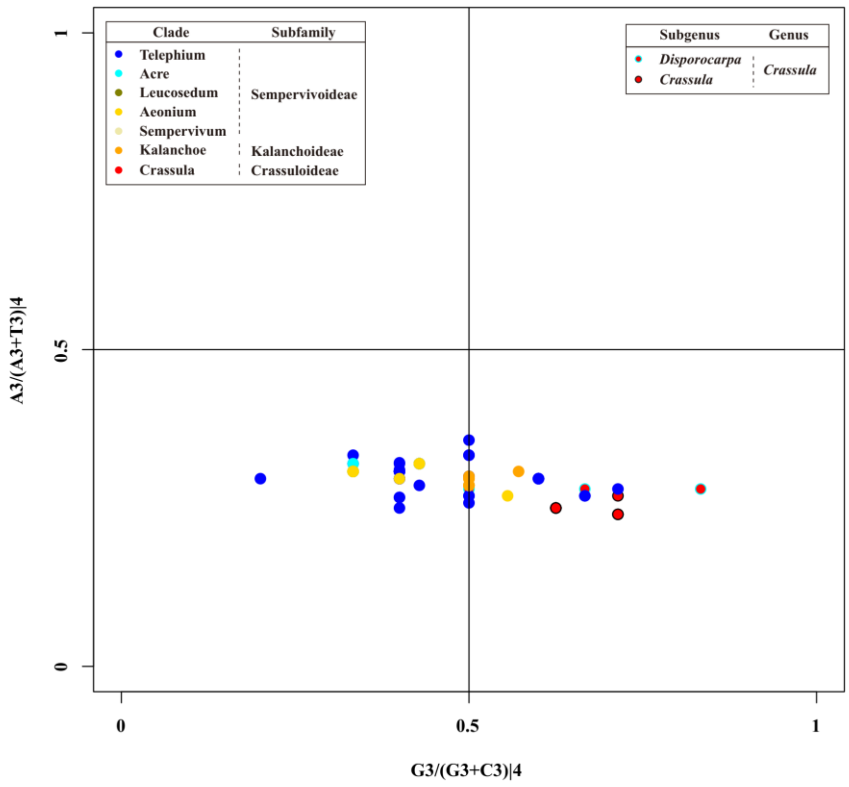
Clade
Subfamily
Telephium
Acre
Leucosedum
Aeonium
Sempervivum
Kalanchoe
Crassula
Sempervivoideae
Kalanchoideae
Crassuloideae
Subgenus
Genus
Disporocarpa
Crassula
Crassula
A3/(A3+T3)|4
G3/(G3+C3)|4
0
0.5
1
0
0.5
1

rps11:

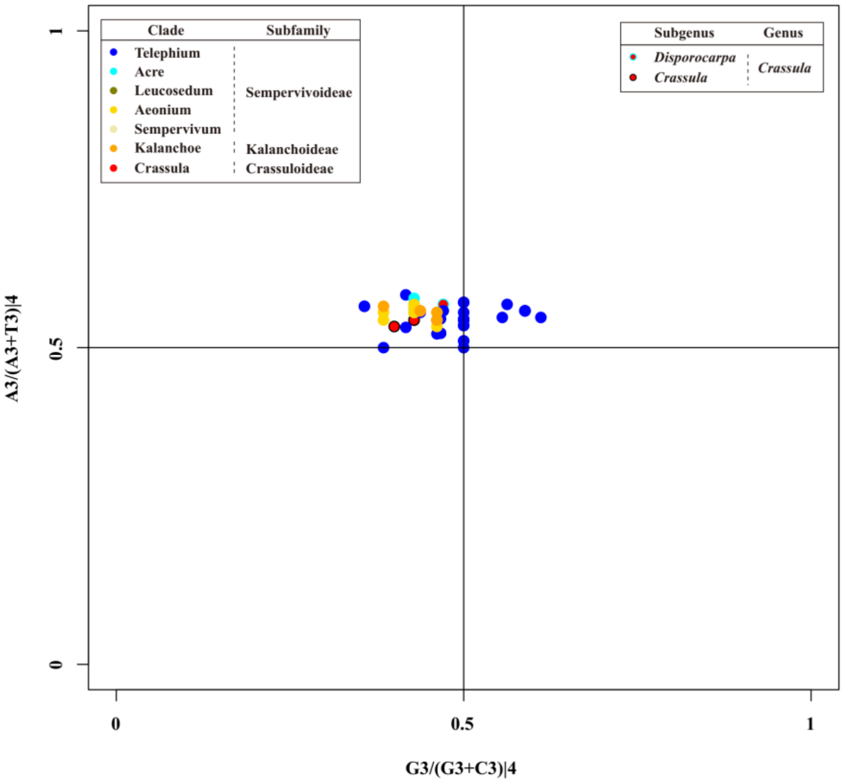

Clade
Subfamily
Telephium
Acre
Leucosedum
Aeonium
Sempervivum
Kalanchoe
Crassula
Sempervivoideae
Kalanchoideae
Crassuloideae
Subgenus
Genus
Disporocarpa
Crassula
Crassula
A3/(A3+T3)|4
G3/(G3+C3)|4
0
0.5
1

rps12:

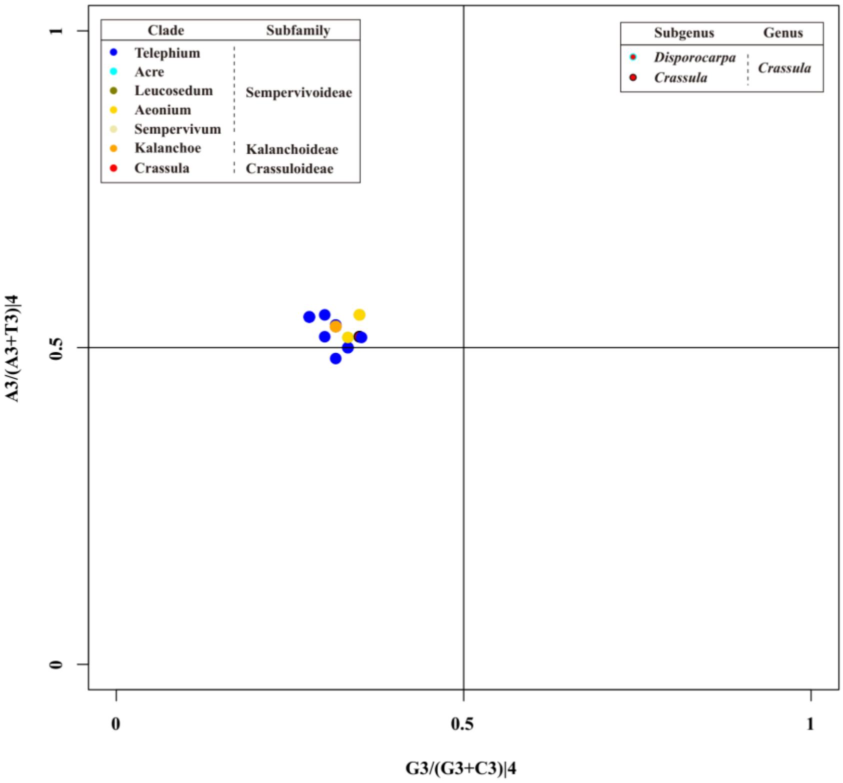

Clade
Subfamily
Telephium
Acre
Leucosedum
Aeonium
Sempervivum
Kalanchoe
Crassula
Sempervivoideae
Kalanchoideae
Crassuloideae
Subgenus
Genus
Disporocarpa
Crassula
Crassula
A3/(A3+T3)|4
G3/(G3+C3)|4
0
0.5
1

rps14:

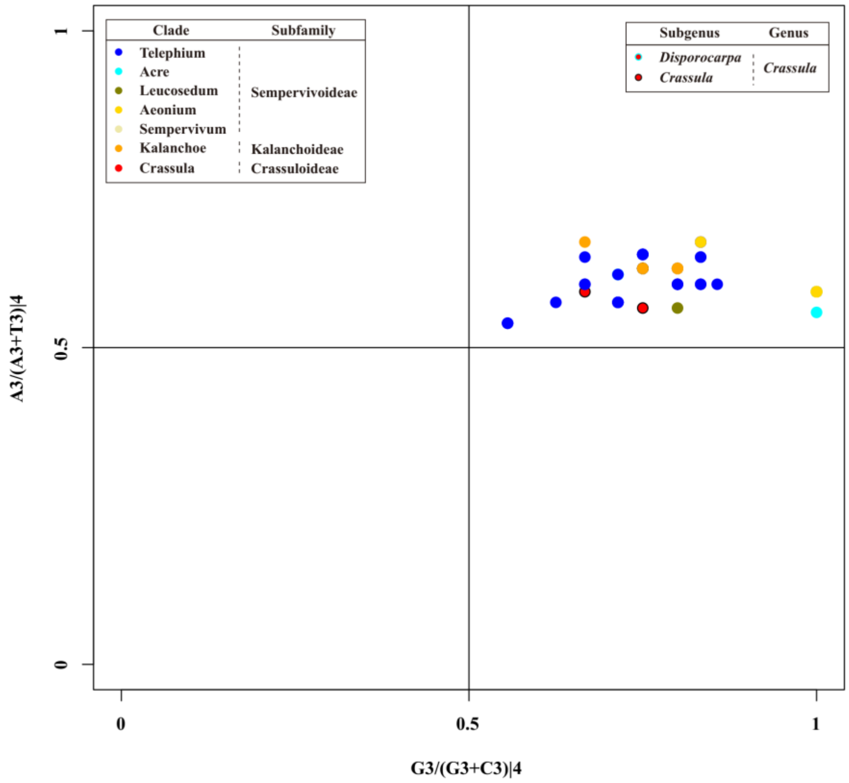
Clade
Subfamily
Telephium
Acre
Leucosedum
Aeonium
Sempervivum
Kalanchoe
Crassula
Sempervivoideae
Kalanchoideae
Crassuloideae
Subgenus
Genus
Disporocarpa
Crassula
Crassula
A3/(A3+T3)|4
G3/(G3+C3)|4
0
0.5
1
0
0.5
1

rps18:

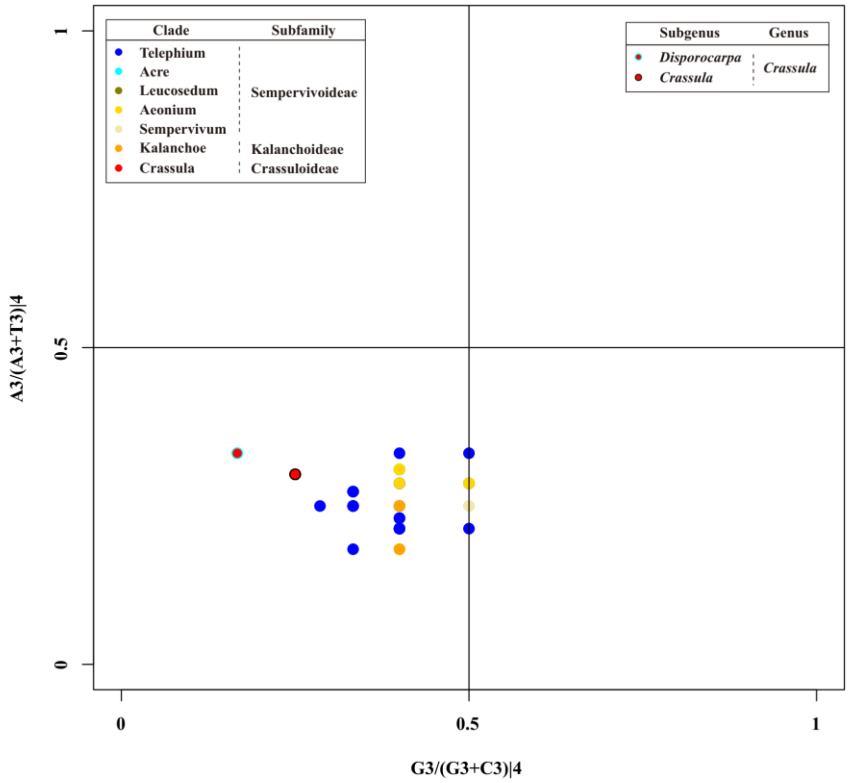
Clade
Subfamily
Telephium
Acre
Leucosedum
Aeonium
Sempervivum
Kalanchoe
Crassula
Sempervivoideae
Kalanchoideae
Crassuloideae
Subgenus
Genus
Disporocarpa
Crassula
Crassula
A3/(A3+T3)|4
G3/(G3+C3)|4
0
0.5
1
0
0.5
1

ycf1:

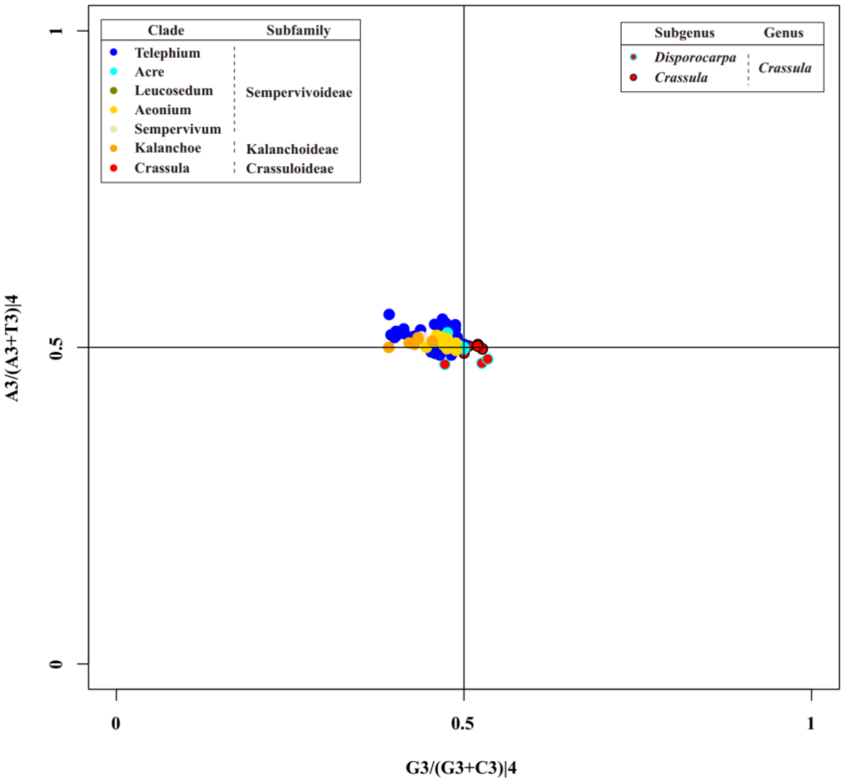

Clade
Subfamily
Telephium
Acre
Leucosedum
Aeonium
Sempervivum
Kalanchoe
Crassula
Sempervivoideae
Kalanchoideae
Crassuloideae
Subgenus
Genus
Disporocarpa
Crassula
Crassula
A3/(A3+T3)|4
G3/(G3+C3)|4
0
0.5
1

ycf2:

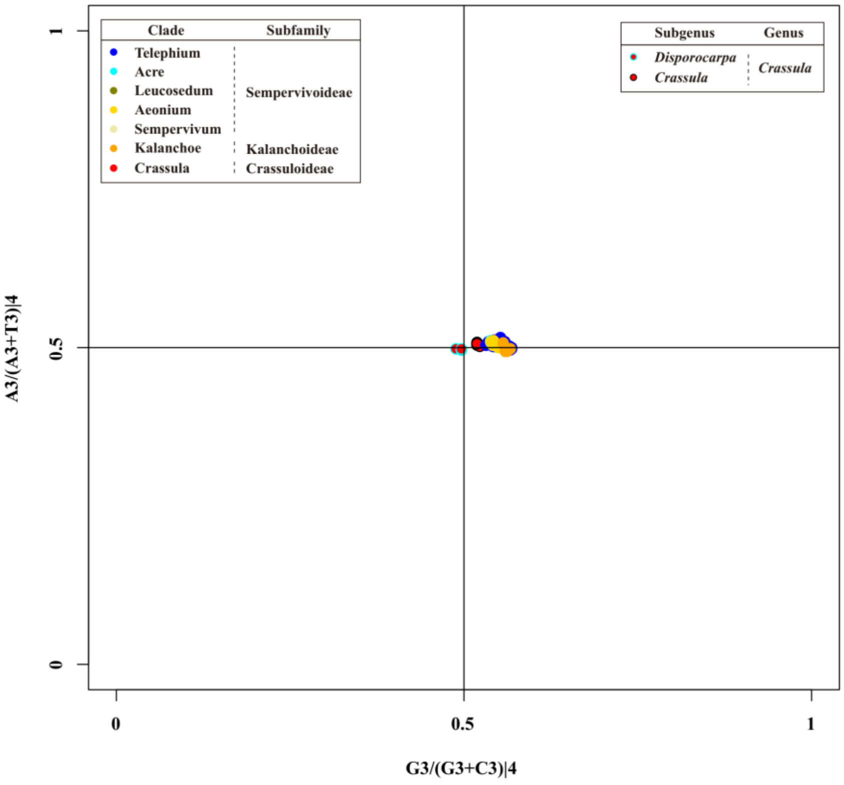

Clade
Subfamily
Telephium
Acre
Leucosedum
Aeonium
Sempervivum
Kalanchoe
Crassula
Sempervivoideae
Kalanchoideae
Crassuloideae
Subgenus
Genus
Disporocarpa
Crassula
Crassula
A3/(A3+T3)|4
G3/(G3+C3)|4
0
0.5
1
